# Correlations among different parts, growth dynamics, anatomical structure and quality traits of *Dendrocalamus brandisii* young shoots

**DOI:** 10.3389/fpls.2026.1982727

**Published:** 2026-10-22

**Authors:** Honglang Luo, Lixin Huang, Song Guo, Xiuhua Zhang, Yuan Wang, Deming Lu, Qian Lu, Zailiu Li

**Affiliations:** 1 Guangxi Colleges and Universities Key Laboratory for Cultivation and Utilization of Subtropical Forest Plantation, Guangxi Key Laboratory of Forest Ecology and Conservation, College of Forestry, Guangxi University, Nanning, China; 2 Guangxi Zhuang Autonomous Region State-owned Qipo Forest Farm, Nanning, China; 3 College of Architectural Arts, Guangxi Arts University, Nanning, China; 4 Nanning Arboretum, Guangxi Zhuang Autonomous Region, Nanning, China

**Keywords:** anatomical structure, *Dendrocalamus brandisii*, edible bamboo shoots, growth dynamics, nutritional components

## Abstract

*Dendrocalamus brandisii* is a high-value horticultural vegetable crop prized for its sweet and tender shoots, yet its stage- and tissue-specific nutrient dynamics remain unclear, limiting standardized harvesting for the fresh-market industry. Here, we combined morphological observation, anatomical analysis, nutrient quantification, and TOPSIS comprehensive evaluation to elucidate the growth pattern, anatomical characteristics, and distribution of quality-related components in young shoots. The edible period lasted 25 days from emergence to the point of inedibility, and height growth followed a slow-then-fast pattern that was divided into four stages (I–IV). Anatomically, cell division dominated in Stages I–II, whereas cell elongation dominated in Stages III–IV. Nutrient contents exhibited significant spatiotemporal heterogeneity: moisture, vitamin C, and flavonoids were highest in the upper part at Stage I; starch and protein peaked in the lower part at Stages II and III, respectively; soluble sugars, fats, cellulose, and lignin accumulated continuously during Stages III–IV; and total free amino acids as well as sweet amino acids reached their maximum in the upper part at Stage III. Ca, Mg, Fe, Zn, Mn, and Cu contents peaked at Stage I and then declined, whereas K peaked at Stage II and Na showed no clear directional change. Oxalic acid was highest in the middle part at Stage IV, whereas tannin content peaked in the upper part at Stage I and then declined. TOPSIS comprehensive ranking confirmed Stage III (19–22 d) as the optimal harvest window, with the upper and middle segments as premium raw materials for fresh consumption. This study reveals the anatomical-metabolic basis of shoot quality and provides a scientific basis for both large-scale *D. brandisii* cultivation and precision harvesting in bamboo vegetable production. These findings offer practical guidance for timing the harvest and selecting the premium edible portions of *D. brandisii* shoots, thereby enhancing the commercial value and economic returns of fresh bamboo shoots.

## 1 Introduction

Bamboos are characterized by rapid growth and canopy closure, short rotation periods, and high productivity, offering substantial economic, ecological, and social benefits (Du et al., 2018). Bamboo shoots are young buds or tender stems sprouting from the underground rhizomes of bamboo plants (Wang et al., 2020). They grow rapidly and are rich in dietary fiber, protein, amino acids, mineral elements, and vitamins, while being low in cholesterol and fat. With their delicious taste, bamboo shoots are regarded as a traditional forest vegetable in China, East Asia, and Southeast Asia, often praised as a “treasure among vegetables” (Choudhury et al., 2012).

The nutrient composition of bamboo shoots changes dynamically during vertical elongation (Guo et al., 2023). Different bamboo species exhibit distinct growth rhythms and nutrient dynamics. For instance, *Dendrocalamus latiflorus*, a sympodial bamboo species cultivated for both young shoots and timber, displays a “slow-then-fast” vertical growth pattern, with an edible period of 21 days. Based on a comprehensive evaluation of sugars, protein, and fiber, its optimal harvesting height is 20–40 cm (Huang et al., 2025b). For *Chimonobambusa tumidissinoda*, an amphipodial mixed bamboo species, shoots exhibit the optimal nutritional quality, taste, and economic value at a height of 10–20 cm (Yang et al., 2015). In the monopodial scattered bamboo *Phyllostachys bambusoides*, shoots harvested at <10 cm in height display the optimal nutritional quality (Gan et al., 2013). For spring shoots of *Phyllostachys edulis*, the nutritional and flavor quality is highest immediately after soil emergence (Guo et al., 2023). Therefore, systematic investigation of bamboo shoot growth dynamics and nutrient variation during development is critical for determining the optimal harvesting window and establishing a scientific basis for rational shoot harvesting.

In addition, bamboo shoots are structurally composed mainly of apical meristems and intercalary meristems of internodes. Apical meristems originate from primordial meristematic cells and their derivatives, while intercalary meristems of internodes are differentiated from apical meristems (Xiong et al., 1980). Apical meristems dominate the elongation of the growth axis and the morphogenesis of lateral buds, whereas the rapid growth of bamboo shoots mainly relies on cell division and elongation in intercalary meristems, especially the significant elongation of longitudinal internodes. Changes in cell length and nuclear number in tissues directly reflect cell elongation and division (Chen et al., 2022; Cui et al., 2012). However, the differentiation patterns of cells and tissues vary among bamboo species, and even differ among growth stages of the same species. For instance, anatomical results of *Bambusa intermedia* at different growth stages reveal significant differences in the number and length of fiber cells, as well as the differentiation and maturity of parenchyma cells and vascular bundles (He et al., 2017). For *P. edulis*, internode elongation is dominated by cell division and an increase in cell number at the initial stage, and gradually transitions to cell elongation as the main driving force in the middle and late stages (He et al., 2013). Thus, cell division and elongation are key factors influencing the growth and development of bamboo shoots. Anatomical observation of bamboo shoots at different growth stages can reveal their growth and development patterns and clarify tissue differentiation characteristics. This is of great significance for revealing the dynamics of nutrient accumulation.

During shoot growth, the contents of protein, vitamins, dietary fiber, and lignin continuously change (Huang et al., 2025a), directly affecting nutritional value and taste (Wei et al., 2025). Studies have shown that various substances in bamboo shoots change dynamically with growth stage. During underground development, shoots accumulate soluble sugars to meet the energy demands of respiration and structural differentiation (Martínez-Vilalta et al., 2016). After emergence, during the rapid growth phase, soluble sugars are often mobilized for energy, with their content decreasing in some bamboo species (Zhang et al., 2021), and protein accumulation also declines (Yang et al., 2015). In the middle and late stages, cellulose and lignin contents increase significantly (Tao et al., 2020). Additionally, nutrients exhibit spatial heterogeneity within the shoot. Protein levels are lower at the base than in the middle and upper sections (Lin et al., 2018). Contents of protein and starch are higher in internodes than in nodes (Fan et al., 2023). In winter shoots of *P. edulis*, total sugar, protein, and flavonoids in the flesh are significantly higher than in the shoot tip and sheath (Huang et al., 2014). Core nutrient contents vary markedly among bamboo species, which is a key factor driving differences in nutritional value and quality. For example, Chen et al. (2023) found that *Chimonobambusa quadrangularis* shoots have high protein content, while *Dendrocalamopsis oldhami* shoots are notable for their high crude fiber content. Tong et al. (2013) measured the nutrient components of *Phyllostachys acuta* shoots and found that they were higher than those of *P. edulis* spring shoots, indicating superior basic nutritional value. Wu et al. (2022) studied the nutrients of 11 bamboo species and reported that *C. utilis* shoots are characterized by high protein and low fat, *D. latiflorus* shoots by high sugar, and *C. hirtinoda* and *C. angustifolia* shoots have the best overall quality. Thus, significant interspecific differences exist in nutrient contents. Systematic investigation of the nutrient dynamics of *D. brandisii* shoots will therefore precisely identify the optimal nutritional stage, providing a key theoretical basis for selecting the best harvesting period.

*Dendrocalamus brandisii* is a high-value bamboo species belonging to the subfamily Bambusoideae, genus Dendrocalamus. Its shoots are renowned for their sweet, tender, and crisp texture (Chen et al., 2025). Native to Yunnan, it has been introduced to Guangdong, Guangxi, Sichuan, and other regions, and thrives in acidic, fertile soils (Hui et al., 2020). The shoots are rich in protein, dietary fiber, vitamins, and other nutrients, with low caloric content and high yield, making them suitable for fresh consumption as well as various culinary and processing applications (Hui et al., 2022; Zhang et al., 2022). Recent research has systematically evaluated the stand biomass and site environmental conditions of *D. brandisii*, revealing the regulatory effects of soil and climatic factors on mature culm productivity and offering theoretical support for regional plantation planning and yield assessment (Wang et al., 2026). Nevertheless, such work mainly focuses on mature bamboo stands, with little attention to shoot development and the quality characteristics of the edible shoots. Previous studies have demonstrated that the contents of protein, total sugar, crude fiber, and mineral elements in young *D. brandisii* shoots vary significantly among different provenances, with the Lincang provenance exhibiting the best comprehensive nutritional quality (Shi et al., 2025). In terms of postharvest preservation, low-temperature plasma treatment has been shown to delay lignification and extend storage life (Yang et al., 2026b). A recent comparative study on the quality of four bamboo shoot species further confirmed that *D. brandisii* shoots have the highest overall acceptability, and that flavor differences are mainly attributable to amino acids, fats, carbohydrates, fiber, and bitter compounds (Zhang et al., 2025b). However, the growth dynamics and nutrient accumulation patterns of young *D. brandisii* shoots remain unclear. Whether nutrient composition differs across growth stages and among parts within the same stage is also unknown. This knowledge gap hinders scientific decision-making regarding the optimal harvesting time and portion, thereby restricting the standardized production of the *D. brandisii* shoot industry. With the rapid expansion of *D. brandisii* cultivation area across southern China and the increasing market demand for high-quality sweet shoots, addressing this research gap has become an urgent priority for industrial development. Therefore, this study focused on young *D. brandisii* shoots cultivated in Nanning, integrating morphological observation, anatomical analysis, and dynamic nutrient monitoring to systematically elucidate their growth patterns, anatomical structural changes, and nutrient accumulation dynamics. Specifically, this study addressed the following scientific questions: (1) the growth dynamics of young *D. brandisii* shoots and the classification of their growth stages; (2) the morphological changes in internode cells at different stages and their relationships with cell division, differentiation, elongation, and height growth; (3) the spatiotemporal dynamics of nutrients and anti-nutritional substances across different parts and growth stages; and (4) the optimal nutritional stage and parts, as identified by multidimensional TOPSIS comprehensive evaluation. This study aimed to elucidate, at the cellular and metabolite levels, the key stages and parts responsible for quality formation in *D. brandisii* shoots, thereby providing a scientific basis for cultivation and harvesting, theoretical support for the promotion and planting of this species, and guidance for the sustainable development of the *D. brandisii* industry.

## 2 Materials and methods

### 2.1 Experimental site

The study was conducted at the Lixin Substation of State-owned Qipo Forest Farm, Wuxu Town, Nanning, Guangxi Zhuang Autonomous Region, China (22°41′24″ N, 108°12′36″ E). The experimental site has a subtropical monsoon climate, with an annual average temperature of 21.8 °C, annual average sunshine duration of 1480.4 h, and a frost-free period of 270–300 d (**Supplementary Figure S1**). The rainy season spans from April to September, with an average annual precipitation of 1453.4 mm. The experimental forest is situated at an altitude of ca. 300 m and on a slope of ca. 30°. The *D. brandisii* plantation covers 0.43 hm^2^ and was established in 2019 at a spacing of 4 m × 2 m. Throughout the study, the plantation was neither artificially irrigated nor chemically fertilized and was managed only by routine manual weeding, following the conventional local production practice for this bamboo shoot stand. The soil is predominantly strongly acidic lateritic red soil with minimal human disturbance. A recent soil survey of Qipo Forest Farm reported that the topsoil (0–20 cm) is strongly acidic (pH 4.37), with an organic matter content of 25.50 g/kg, a total nitrogen content of 1.25 g/kg, an available phosphorus content of 1.18 mg/kg, and an available potassium content of 52.75 mg/kg (Lin et al., 2024). The site has abundant sunshine and rainfall as well as a mild climate, rendering it suitable for the growth of *D. brandisii* (Tang et al., 2022).

### 2.2 Plant materials

During the shooting season, six representative sample plots (10 m × 10 m) were randomly established in the experimental stand to monitor the growth of newly emerged young shoots. In each plot, 15 healthy, intact, and pest-free young *D. brandisii* shoots were randomly selected, tagged, and numbered, yielding a total of 90 shoots that were monitored daily for growth. Shoot height and basal diameter were measured daily from emergence (defined as the shoot tip protruding 2–3 cm above the ground) until the shoots became inedible (judged as the stage when the internal tissues had become so fibrous and lignified that a fingernail could no longer be pressed transversely into them) (Hui et al., 2020). Growth data were analyzed using the sequential clustering (optimal segmentation) method, which partitions the ordered samples (daily cumulative height values of the edible period) into classes by minimizing the within-class error function; the optimal number of classes was determined by the minimum-error criterion, yielding four growth stages (Zhou, 1999). At each stage, six healthy young shoots were sampled from each of the six plots, giving 36 shoots per stage (6 plots × 6 shoots); the six shoots from each plot were pooled into one composite sample, and the six composite samples (one per plot) served as six biological replicates (n = 6). After soil removal and sheath stripping, the shoot bases (culm bases) were excised to obtain the edible fraction. The edible fraction was then cut transversely into three segments of equal length, designated as the upper (apical), middle, and lower (basal) parts (Li et al., 2024; **Supplementary Figures S2A, B**). Each segment was cut into 1 cm^3^ cubes. One subsample was fixed in FAA fixative (formaldehyde: glacial acetic acid: 70% ethanol = 5:5:90, v/v) for paraffin sectioning and anatomical examination (**Supplementary Figures S2C, D**). Another subsample was stored at −80 °C for subsequent assays of moisture, vitamin C, soluble sugar, starch, free amino acids, oxalic acid, and tannins. The remaining subsample was oven-dried at 105 °C for 30 min to inactivate enzymes, further dried at 65 °C to constant weight, ground, and sieved through a 60-mesh sieve to obtain dry powder.

### 2.3 Index determination

#### 2.3.1 Shoot growth status

From the day of shoot emergence (when the shoot tip protruded 2–3 cm above the ground), shoot height was measured daily at 9:00 a.m. using a tape measure (accurate to 0.1 cm). The basal diameter of the aboveground part of the shoot was measured using a vernier caliper (accurate to 0.01 cm); two perpendicular directions were measured for each shoot, and the average value was taken.

#### 2.3.2 Anatomical structure

The morphological and anatomical characteristics of young *D. brandisii* shoots were examined using a paraffin sectioning method modified from Li (1987). FAA-fixed samples were dehydrated through a graded ethanol series, cleared with TO (a bio-clearing agent), and embedded in paraffin. Sections of 8–12 μm thickness were cut using a microtome (Leica RM 2245, Germany). After deparaffinization, staining, and mounting, the sections were observed under a microscope (Zeiss Axio Imager Z2, Germany). Both longitudinal and transverse sections were prepared for each sample, with six sections per orientation. During observation, each section was first examined at 10×10 magnification to assess the overall structure, and three representative fields of view were selected per section, focusing on the distribution and arrangement of the epidermis, ground tissue, and vascular bundles. Subsequently, the same fields were observed at 10×40 magnification to evaluate cytological features, including the division status of parenchyma cells, the size and distribution of cell nuclei, the elongation of fiber cells, the degree of development of vascular bundles, and the type and inner diameter of vessel elements. Representative images were captured for each field of view.

#### 2.3.3 Nutrients and chemical components

Samples from the upper, middle, and lower parts of young shoots at different growth stages were randomly collected for nutrient determination. The specific indices measured included moisture content, soluble sugar, starch, protein, fat, vitamin C, cellulose, lignin, flavonoids, free amino acids, mineral elements, and anti-nutritional substances (oxalic acid and tannins). Each index was determined on six biological replicates (n = 6), with three technical replicates per composite sample. All nutrient contents are expressed on a dry weight (DW) basis unless otherwise stated; moisture, vitamin C, oxalic acid, and tannin are expressed on a fresh weight (FW) basis.

##### 2.3.3.1 Moisture content

Moisture content was determined gravimetrically according to the national standard GB 5009.3-2016 (National Health and Family Planning Commission of the People's Republic of China, 2016). Fresh samples were dried at 105 °C to constant weight. Moisture content was calculated as (fresh weight – dry weight)/fresh weight × 100%.

##### 2.3.3.2 Soluble sugar and starch content

Soluble sugar and starch contents were determined using the anthrone-sulfuric acid colorimetric method (Gao and Cai, 2018). Samples were extracted with deionized water at 80 °C for 60 min. For soluble sugar determination, the extract was reacted with anthrone reagent at 90 °C for 15 min and absorbance was measured at 625 nm. For starch determination, the residual pellet after extraction was hydrolyzed with 6 mol/L HCl at 100 °C for 30 min, neutralized, and then subjected to the same colorimetric procedure. A sucrose standard curve was used for quantification.

##### 2.3.3.3 Protein content

Protein content was determined by the Coomassie Brilliant Blue method (Gao and Cai, 2018). Fresh samples (1.0 g) were extracted with 80 mL deionized water under ultrasound at 40 °C for 15 min. After centrifugation at 4000 × g and 4 °C for 15 min, 0.5 mL of supernatant was mixed with 5 mL Coomassie Brilliant Blue working solution (0.1 g dye in 50 mL 95% ethanol and 100 mL 85% phosphoric acid, diluted to 1 L). After 2 min at room temperature, absorbance was measured at 595 nm. A standard curve was prepared using bovine serum albumin (BSA, 0–0.1 mg/mL).

##### 2.3.3.4 Fat content

Fat content was determined by the Soxhlet extraction method (Gao and Cai, 2018). Dried samples (1.0 g) were placed in a cellulose thimble and extracted with anhydrous ether for 6–10 h at a reflux rate of 6–8 cycles per hour. After ether recovery, the receiving flask was dried at 105 °C to constant weight (difference ≤0.002 g) and weighed.

##### 2.3.3.5 Vitamin C content

Vitamin C content was determined by the 2,6-dichloroindophenol titration method (Gao and Cai, 2018). Fresh samples (5.0 g) were ground with 20 mL 2% oxalic acid, diluted to 100 mL with 1% oxalic acid, and filtered. An aliquot (20 mL) of the filtrate was titrated with standardized 2,6-dichloroindophenol solution to a pink endpoint that persisted for 15 s. All operations were performed under light-free conditions at 20–25 °C. Vitamin C content was calculated based on the volume of titrant consumed and expressed as mg·100g^-1^.

##### 2.3.3.6 Cellulose content

Cellulose content was determined by the anthrone-H_2_SO_4_ colorimetric method (An et al., 2016). Dried samples (0.5 g) were refluxed with 10 mL 80% ethanol at 80 °C for 30 min to remove soluble sugars, followed by centrifugation. The precipitate was treated with 10 mL 1 mol/L NaOH at 80 °C for 30 min to remove hemicellulose, washed with deionized water to neutrality, and dissolved in 5 mL concentrated H_2_SO_4_ on an ice bath. An aliquot (1 mL) of the diluted solution was mixed with 5 mL anthrone reagent, heated in a boiling water bath for 15 min, and absorbance was measured at 625 nm. A standard curve was prepared using glucose.

##### 2.3.3.7 Lignin content

Lignin content was determined by the acid hydrolysis method (Gao and Cai, 2018). Dried samples (0.5 g) were extracted with deionized water for 24 h to remove impurities and dried at 70 °C. The residue was treated with 3 mL 72% H_2_SO_4_ at room temperature for 2 h with mixing every 15 min, diluted with 84 mL deionized water to 4% H_2_SO_4_, and hydrolyzed at 121 °C for 1 h. The hydrolysate was filtered, and the solid residue was washed with deionized water to neutrality, dried at 105 °C to constant weight, and weighed.

##### 2.3.3.8 Flavonoid content

Flavonoid content was determined by the NaNO_2_-Al(NO_3_)_3_ colorimetric method (Gao and Cai, 2018). Samples (1.0 g) were extracted with 100 mL 70% ethanol containing a small amount of CaCO_3_ for 6–8 h, followed by concentration under reduced pressure. The concentrate was washed with diethyl ether and diluted to 100 mL with 70% ethanol. One mL of the sample solution was sequentially mixed with 0.3 mL 5% NaNO_2_ (6 min), 0.3 mL 10% Al(NO_3_)_3_ (6 min), and 2 mL 4% NaOH, and absorbance was measured at 510 nm. A standard curve (0–50 μg/mL) was prepared using rutin.

##### 2.3.3.9 Amino acid content

Free amino acids were determined according to GB/T 30987-2020 (State Administration for Market Regulation and Standardization Administration of China, 2020). Dried samples (1.0 g) were extracted with 80% ethanol at 65 °C for 1 h with shaking. After centrifugation, the supernatant was collected, and the residue was re-extracted once. The combined extract was filtered through a 0.22 μm membrane and subjected to automatic pre-column derivatization with o-phthaldialdehyde (OPA) and 9-fluorenylmethyl chloroformate (FMOC). Separation was performed on an HPLC system equipped with a C18 column using a gradient elution of sodium acetate buffer (pH 6.5) and acetonitrile-methanol-water, with fluorescence detection (Ex: 340 nm, Em: 450 nm). A mixed standard containing 17 amino acids was used for qualitative and quantitative analysis.

##### 2.3.3.10 Mineral elements

Mineral elements were determined by inductively coupled plasma mass spectrometry (ICP-MS) according to the national standard GB 5009.268-2025 (National Health Commission of the People's Republic of China and State Administration for Market Regulation, 2025). Dried samples (0.5 g) were digested with 5 mL HNO_3_ (ultrapure grade) and 2 mL 30% H_2_O_2_ in a microwave digestion system (program: 120 °C/5 min, 160 °C/10 min, 190 °C/20 min). The digest was diluted to 50 mL with ultrapure water. The contents of K, Ca, Mg, Na, Cu, Fe, Zn, and Mn were determined by ICP-MS (RF power 1550 W, carrier gas flow 1.05 L/min, spray chamber temperature 2 °C), with rhodium (Rh) or indium (In) as internal standards to correct matrix effects. External calibration curves were prepared using mixed standard solutions.

##### 2.3.3.11 Anti-nutritional substances

Oxalic acid content was determined using a colorimetric method (Duan et al., 2007). Fresh samples (2.0 g) were extracted with 20 mL 0.5 mol/L HCl at 80 °C for 30 min, diluted to 50 mL, and filtered. One mL of the filtrate was mixed with 2.0 mL 0.5 mol/L HCl, 1.0 mL 10% zinc acetate, and 1.0 mL 0.5 mol/L NaOH, heated in a boiling water bath for 5 min, then mixed with 2.0 mL 0.5% 1,10-phenanthroline and 1.0 mL 5% FeCl_3_, and absorbance was measured at 510 nm. A standard curve (0–100 μg/mL) was prepared using oxalic acid.

Tannin content was determined using the Folin-Denis spectrophotometric method (Gao and Cai, 2018). Fresh samples (2.0 g) were extracted with 20 mL 70% acetone under ultrasonication at 40°C for 30 min, followed by centrifugation at 4000 × g for 10 min. The supernatant was collected, and the residue was re-extracted once. The combined extract was diluted to 50 mL. One mL of the sample solution was mixed with 5.0 mL Folin-Denis reagent and 10 mL saturated Na_2_CO_3_, diluted to volume with ultrapure water, incubated at room temperature in the dark for 30 min, and absorbance was measured at 760 nm. A standard curve (0–100 μg/mL) was prepared using tannic acid.

### 2.4 Data analysis

Data were statistically analyzed using Excel 2021, Python 3.12, SPSS 26.0, and SPSSAU. Before formal analysis, the normality of the data distribution was assessed using the Shapiro-Wilk test, and the homogeneity of variances was evaluated using Levene’s test. All variables met the assumptions of normality and homoscedasticity (*P > 0.05*). Analyses of variance (ANOVA) were performed to evaluate the effects of growth stage and shoot part on the measured parameters, with post-hoc multiple comparisons conducted using Duncan’s new multiple range test at *P = 0.05* and *P = 0.01*. Graphs were generated using Origin 2021, and annotations on section images were added using ImageJ. To identify the optimal nutritional stage and parts, the TOPSIS (Technique for Order Preference by Similarity to Ideal Solution) comprehensive evaluation method was applied for ranking (Lei and Qiu, 2016). Indicator weights were determined by the entropy-weight method, and the relative closeness (Ci) was used to rank the growth stages and parts.

## 3 Results

### 3.1 Changes in growth patterns

Daily growth of young *D. brandisii* shoots was monitored from emergence to inedibility during the peak shooting period in Nanning. As growth progressed, the external sheaths and internal tissues of the shoot gradually changed color (**Figure 1**). The sheaths turned from reddish-brown to yellowish-green (**Figure 1A**), while the upper part of the shoot changed from initially white to yellowish-green, and the base gradually shifted from white to brown. When growth exceeded 25 days, the shoots became highly fibrous and lignified, and most of the internal tissues turned green (**Figure 1A-a**), rendering them inedible. During the edible period, shoot height growth followed a “slow-then-fast” trend, lasting a total of 25 days (**Figure 1B**). Using sequential clustering (optimal segmentation), the edible growth period was divided into four stages: Stage I (1–11 d), with an average daily height increment of 1.72 cm and a mean shoot height of 10.95 cm; Stage II (12–18 d), with an average daily height increment of 3.15 cm and a mean shoot height of 31.68 cm; Stage III (19–22 d), with an average daily height increment of 5.60 cm and a mean shoot height of 55.51 cm; Stage IV (23–25 d), with an average daily height increment of 8.23 cm and a mean shoot height of 80.27 cm (**Figure 1B**). The average daily increments in Stages III and IV were 3.3 and 4.78 times that of Stage I, respectively. In addition, the basal diameter of young *D. brandisii* shoots increased from 55.17 mm at emergence to 131.24 mm by the end of Stage IV. The basal diameter growth rate exhibited a “slow-fast-slow” pattern across stages: slow in Stage I, rapid in Stage II and early Stage III, followed by a marked deceleration from approximately 21 days after emergence, becoming relatively flat in Stage IV (**Figure 1C**).

**Figure 1:**
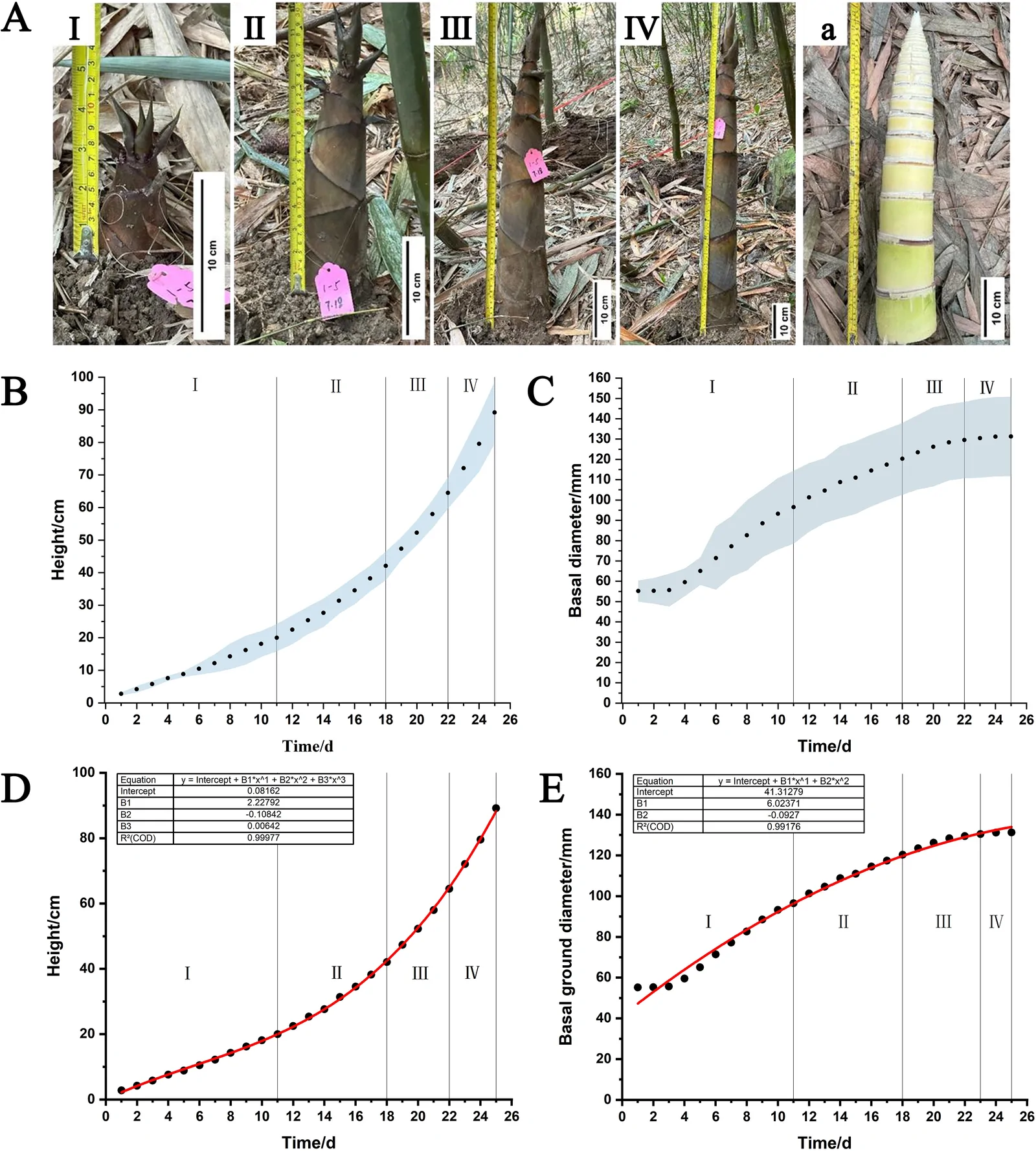
Morphological dynamics and growth models of *D. brandisii* shoots at different developmental stages. **(A)** External morphology of shoots at Stage I, II, III and IV; **(a)** shows the internal morphology of shoots on the 26th day of growth; **(B)** Dynamics of shoot height growth; **(C)** Dynamics of basal diameter growth at soil level; **(D)** Model of shoot height growth; **(E)** Model of basal diameter growth.

With the number of days of shoot height growth as the independent variable (x) and the cumulative daily height as the dependent variable (Y), a growth model for young *D. brandisii* shoot height was established, yielding the fitting equation Y = 0.00642x^3^ - 0.10842x^2^ + 2.22792x + 0.08162, R^2^ = 0.999 (**Figure 1D**). With the number of days of basal diameter growth as the independent variable (x) and the cumulative daily basal diameter as the dependent variable (Y), a growth model for basal diameter was established, yielding the fitting equation Y = -0.0927x^2^ + 6.02371x + 41.31279, R^2^ = 0.992 (**Figure 1E**). Both models provided a good fit and accurately described the dynamics of shoot height and basal diameter growth.

### 3.2 Anatomical structure observation

The cell morphology of internodes of young *D. brandisii* shoots exhibited significant spatiotemporal differentiation across different growth stages (**Figures 2**, **3**). At Stage I, the epidermis of the upper internode consisted of small, tightly arranged cells with large nuclei and dense cytoplasm; intercellular spaces were evident in the cortical parenchyma, and fiber cells remained unelongated. In the middle internode, primary phloem as well as pitted and annular vessels had initially formed, and parenchyma cells showed active division. In the lower internode, vascular bundles began to differentiate, and parenchyma cells and fiber cells exhibited an orderly arrangement (**Figures 2A–C**, **3A–F**). During Stages II‐III, fiber cell number increased significantly in all internodes, and fiber cell elongation gradually accelerated. In the upper internode, parenchyma cells still maintained vigorous division, whereas in the middle and lower internodes, nuclei were no longer visible in some parenchyma cells. The lumen diameters of annular vessels (protoxylem) and pitted vessels (metaxylem) gradually increased, and vascular tissues further matured (**Figures 2D–I**, **3G–R**). At Stage IV, cells in the upper internode remained actively dividing. In the middle and lower internodes, cell division had essentially ceased, most cells had completed differentiation, parenchyma cells were densely and regularly arranged, vascular bundle diameters further increased, and vascular tissues were highly mature (**Figures 2J–L**, **3S–X**). This spatiotemporal heterogeneity in cell differentiation led to a gradual transition in shoot tissue structure, providing an anatomical basis for determining the optimal harvest period.

**Figure 2:**
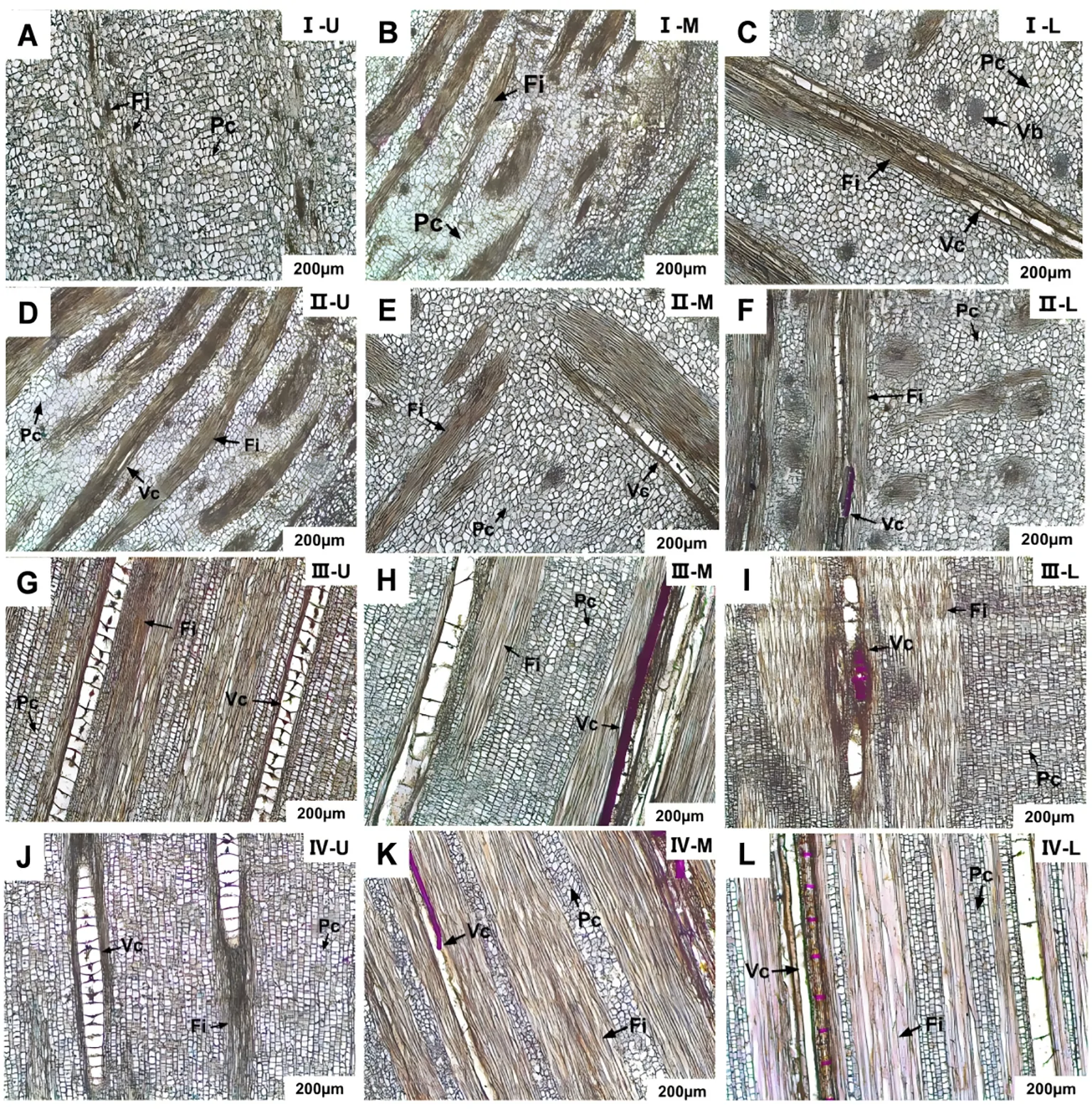
Longitudinal section anatomy of internodes in culm walls of *D. brandisii* shoots across developmental stages and positions. The twelve panels **(A–L)** present the three positions at each of the four developmental stages: **(A)** Stage I, upper; **(B)** Stage I, middle; **(C)** Stage I, basal; **(D)** Stage II, upper; **(E)** Stage II, middle; **(F)** Stage II, basal; **(G)** Stage III, upper; **(H)** Stage III, middle; **(I)** Stage III, basal; **(J)** Stage IV, upper; **(K)** Stage IV, middle; **(L)** Stage IV, basal. (I–IV, Stages I–IV; U, upper; M, middle; L, basal; Ep, epidermis; Pc, parenchyma cells; Fi, fiber cells; Vb, vascular bundle; Vc, vessel elements; Ph, phloem; Px, protoxylem vessel elements; Mx, metaxylem vessel elements).

**Figure 3:**
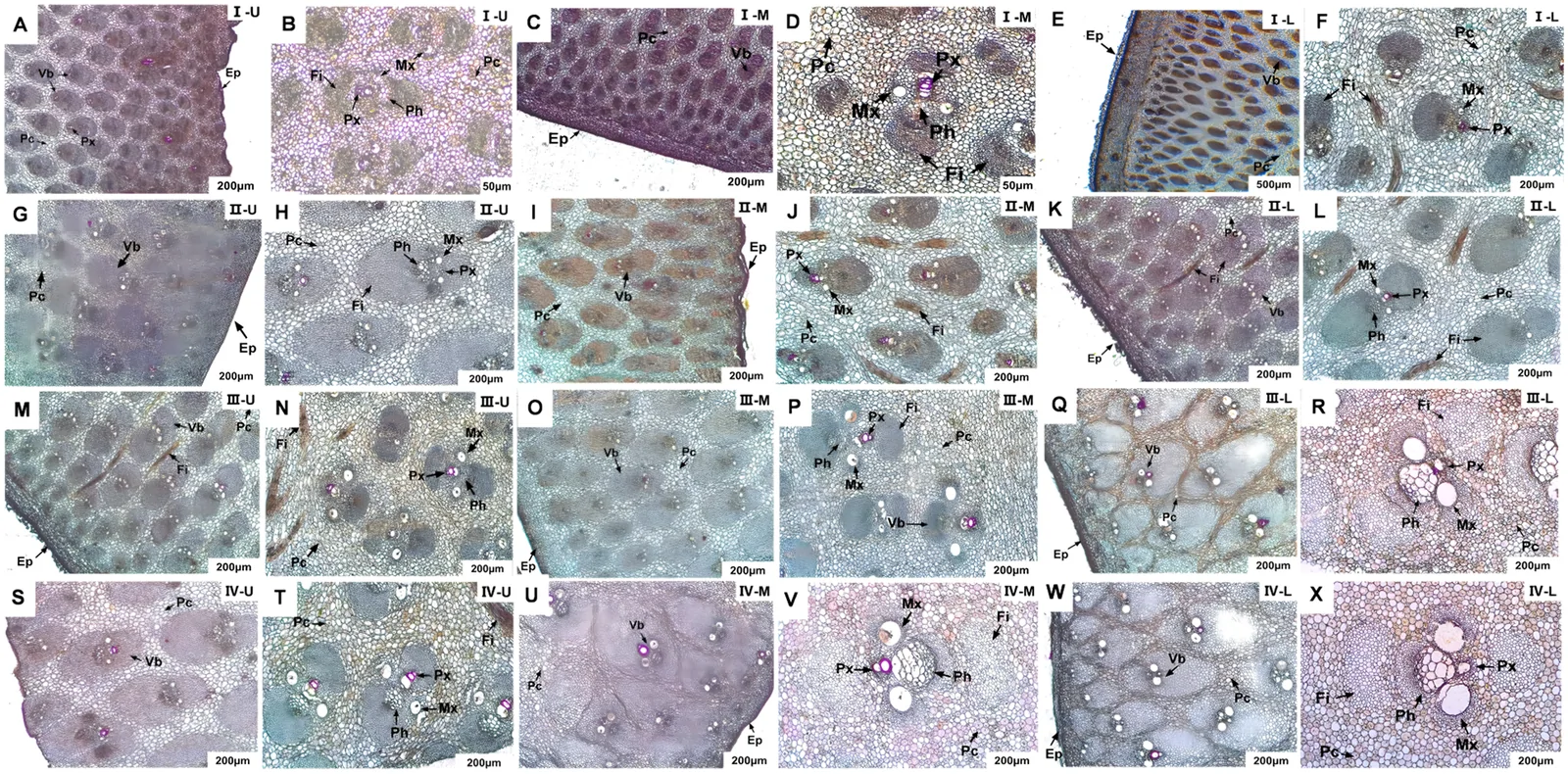
Anatomical structure of internode cross-sections in culm walls of *D. brandisii* shoots at different developmental stages and positions. The twenty-four panels **(A–X)** present two images of each of the twelve stage–position combinations: **(A, B)** Stage I, upper; **(C, D)** Stage I, middle; **(E, F)** Stage I, basal; **(G, H)** Stage II, upper; **(I, J)** Stage II, middle; **(K, L)** Stage II, basal; **(M, N)** Stage III, upper; **(O, P)** Stage III, middle; **(Q, R)** Stage III, basal; **(S, T)** Stage IV, upper; **(U, V)** Stage IV, middle; **(W, X)** Stage IV, basal. (I–IV, Stages I–IV; U, upper; M, middle; L, basal; Ep, epidermis; Pc, parenchyma cells; Fi, fiber cells; Vb, vascular bundle; Vc, vessel elements; Ph, phloem; Px, protoxylem vessel elements; Mx, metaxylem vessel elements).

### 3.3 Nutrient and chemical contents

Variance analysis revealed that the contents of most nutrients and chemical components in young *D. brandisii* shoots differed significantly (*P < 0.01* or *P < 0.05*) among different growth stages as well as among different parts within the same stage (**Figure 4**; **Supplementary Table S2**). The tissue moisture content was highest in the upper part at Stage I, reaching 94.01%, and decreased continuously in all parts as growth progressed, with the lowest value (90.58%) recorded in the lower part at Stage IV. At each stage, moisture content followed a gradient of upper > middle > lower (**Figure 4A**; **Supplementary Table S1**). Soluble sugar content in the upper and lower parts increased continuously during development, reaching 9.12% and 9.81%, respectively, at Stage IV; in the middle part, it showed a “rise-then-fall” trend, peaking at 9.51% at Stage III (**Figure 4B**). Starch content fluctuated dynamically across stages and parts. In the upper part, it first increased and then decreased, peaking at Stage II. In the lower part it increased, decreased, and then increased again, reaching a maximum of 1.00% at Stage II, declining at Stage III, and rising again at Stage IV. The middle part displayed an oscillating decrease–increase–decrease pattern (**Figure 4C**). Protein accumulation showed spatiotemporal heterogeneity: contents in the upper and middle parts increased continuously, reaching 4.35% and 4.15%, respectively, at Stage IV; in the lower part, protein content exhibited a “rise-then-fall” trend, peaking at 4.60% at Stage III (**Figure 4D**). Fat content generally increased, with the highest value (2.78%) detected in the lower part at Stage IV; a gradual upward trend was observed in all three parts (**Figure 4E**). Vitamin C content displayed distinct stage-dependent patterns: in the upper part, it showed a “decrease-then-increase” trend, recovering to 3.44 mg·100g^-1^ at Stage IV; in the middle part, it fluctuated as “decrease-increase-decrease”, peaking at 3.08 mg·100g^-1^ at Stage III; the lower part followed a “rise-then-fall” pattern (**Figure 4F**). Cellulose and lignin, as cell wall skeletal components, both increased continuously with development, reaching maximum values of 5.79% and 7.29%, respectively, in the lower part at Stage IV, and consistently followed a gradient of lower > middle > upper (**Figures 4G, H**). Flavonoid content generally decreased, with the highest value (5.81 mg·g^-1^) observed in the upper part at Stage I (**Figure 4I**).

**Figure 4:**
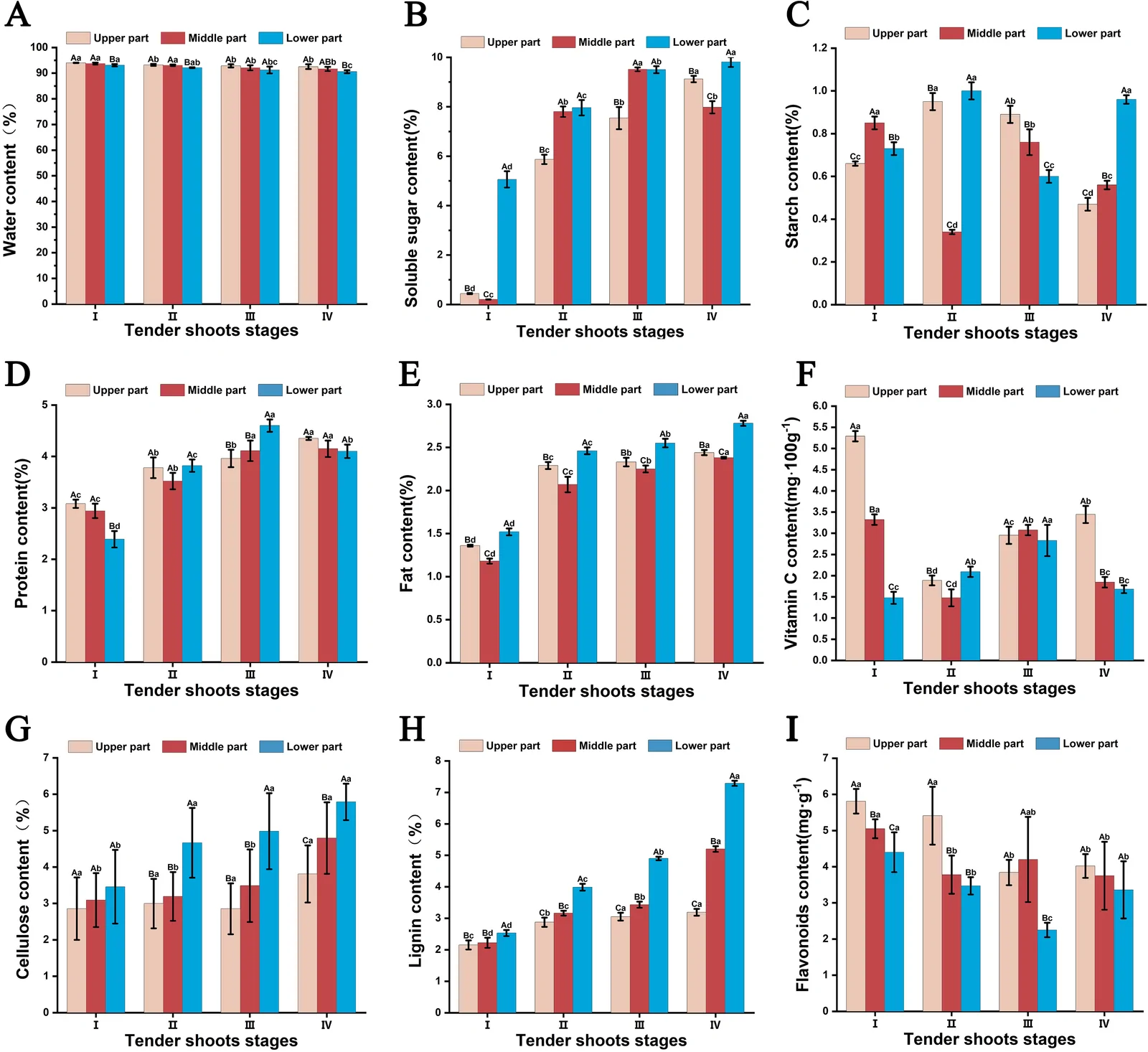
Changes in nutrient content in different parts of young bamboo shoots at different growth stages. (The uppercase letters **(A–I)** in the upper left of the image indicate: **(A)** Moisture content; **(B)** Soluble sugar; **(C)** Starch; **(D)** Protein; **(E)** Fat; **(F)** Vitamin C; **(G)** Cellulose; **(H)** Lignin; **(I)** Flavonoids. Different uppercase letters on the bars indicate significant differences among different parts within the same growth stage (*P<0.05*), while different lowercase letters on the bars indicate significant differences among different growth stages within the same part (*P<0.05*). Values are means of six biological replicates (n = 6).

### 3.4 Free amino acids

Seventeen free amino acids were detected in young *D. brandisii* shoots (**Supplementary Table S3**). The total free amino acid (TAA) content exhibited a “decrease-increase-decrease” trend with shoot growth, with the upper part consistently showing significantly higher levels than the lower part. TAA content peaked in the upper part at Stage III (233.89 mg·100g^-1^) and reached its minimum in the lower part at Stage IV (86.14 mg·100g^-1^). Most free amino acids and their functional categories (essential, sweet, umami, bitter, and aromatic amino acids) showed significant differences across growth stages and parts (P < 0.05 or P < 0.01) (**Supplementary Tables S6, S7**). Correlation analysis (**Figure 5**) revealed that methionine (Met) was negatively correlated with most amino acids, particularly exhibiting highly significant negative correlations with Gly, Val, Ile, Lys, and His (P < 0.01), whereas Thr, Asp, Glu, Ser, Gly, Cys, and Ala were positively correlated with one another (P < 0.01 or P < 0.05).

**Figure 5:**
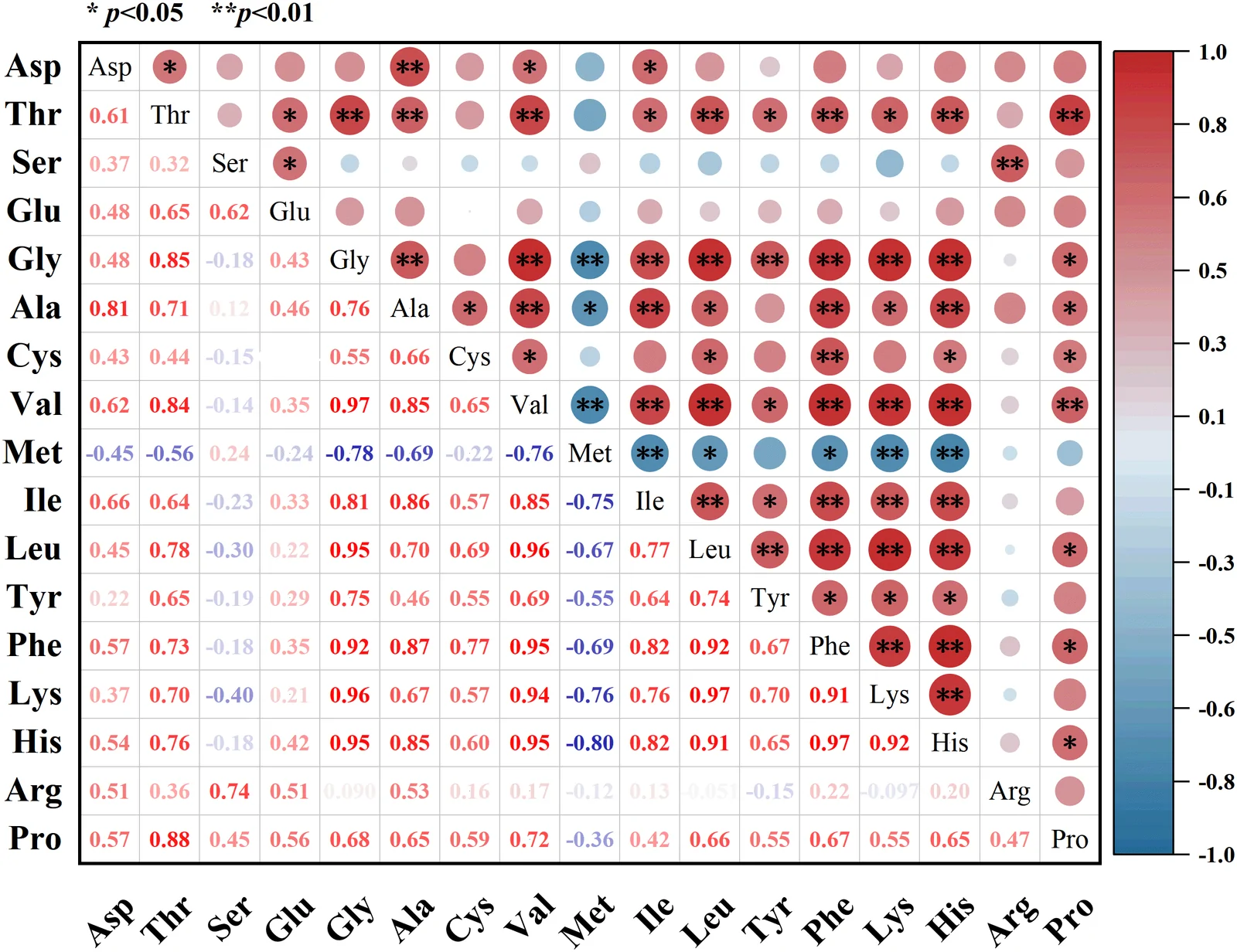
Correlation analysis of free amino acid components in young shoots of *D. brandisii.* (In the image, different tones represent the positive and negative directions of correlation. Red indicates a positive correlation among amino acids, while blue indicates a negative correlation among them. The depth of color represents the absolute value of the correlation coefficient—the darker the color, the larger the absolute value of the correlation coefficient. Correlation analysis was based on data from six biological replicates per stage and part (n = 6). *p<0.05, **p<0.01).

Eight essential amino acids (EAAs; Leu, Ile, Lys, Met, Phe, Thr, Val, His) were detected in young *D. brandisii* shoots. Their total content peaked in the lower part at Stage III (98.03 mg·100g^-1^) and reached its minimum in the lower part at Stage IV (42.35 mg·100g^-1^). The EAA/TAA ratio ranged from 29.03% to 54.55%, with the lower part showing the highest values at Stages I and III, whereas the upper part showed the highest value at Stage IV. Regarding taste-active amino acids, sweet amino acids (Thr, Gly, Ser, Ala, Pro) were highest in the upper part at Stage III (79.32 mg·100g^-1^) and were consistently higher in the upper part than in the middle and lower parts during Stages I–III. Umami amino acids (Glu, Asp) peaked in the middle part at Stage IV (62.50 mg·100g^-1^) and their content in the middle part continuously increased with shoot growth. Bitter amino acids (Ile, His, Val, Met, Leu, Arg) reached their maximum in the lower part at Stage III (96.09 mg·100g^-1^), coinciding with the trend of lignin accumulation. Aromatic amino acids (Tyr and Phe) and the sulfur-containing amino acid Cys were highest in the upper part at Stage I (29.67 mg·100g^-1^ DW) and gradually decreased thereafter (**Supplementary Table S4**).

### 3.5 Mineral elements

Young *D. brandisii* shoots contained four macro-elements (K, Ca, Mg, Na) and four trace elements (Cu, Fe, Zn, Mn). Their contents exhibited significant spatiotemporal heterogeneity across different growth stages and parts (**Supplementary Tables S5, S8**). Among the macro-elements, K content ranged from 22157.67 to 76418.67 mg·kg^-1^, peaking in the lower part at Stage II. From Stages I to III, the distribution pattern was lower > middle > upper, while at Stage IV, the upper part became the highest. The contents of Ca and Mg ranged from 499.40 to 3445.79 mg·kg^-1^ and from 821.03 to 1641.33 mg·kg^-1^, respectively. Both peaked in the middle part at Stage I and gradually decreased with shoot development. In most stages, Ca and Mg contents were higher in the upper or middle part than in the lower part. The Na content was relatively low (348.68–554.30 mg·kg^-1^), peaking in the lower part at Stage I, but showed no clear directional trend. Regarding trace elements, Fe, Zn, and Mn all reached their maximum values in the upper part at Stage I (129.21, 80.79, and 162.43 mg·kg^-1^, respectively) and then gradually decreased as the shoots grew. Similarly, the Cu content was highest in the upper part at Stage I (17.18 mg·kg^-1^) and declined thereafter. Throughout the growth process, the contents of Fe, Zn, and Mn generally followed the distribution pattern of upper > middle > lower.

Correlation analysis (**Figure 6**) revealed that K was significantly negatively correlated with Ca, Mg, Cu, Fe, Zn, and Mn (*P < 0.05* or *P < 0.01*), with the strongest negative correlation observed between K and Zn (*P < 0.01*). Highly significant positive correlations were found between Ca and Mg, Cu and Zn, and Fe and Mn (*P < 0.01*). Sodium (Na) did not show significant correlations with any of the other mineral elements (*P > 0.05*).

**Figure 6:**
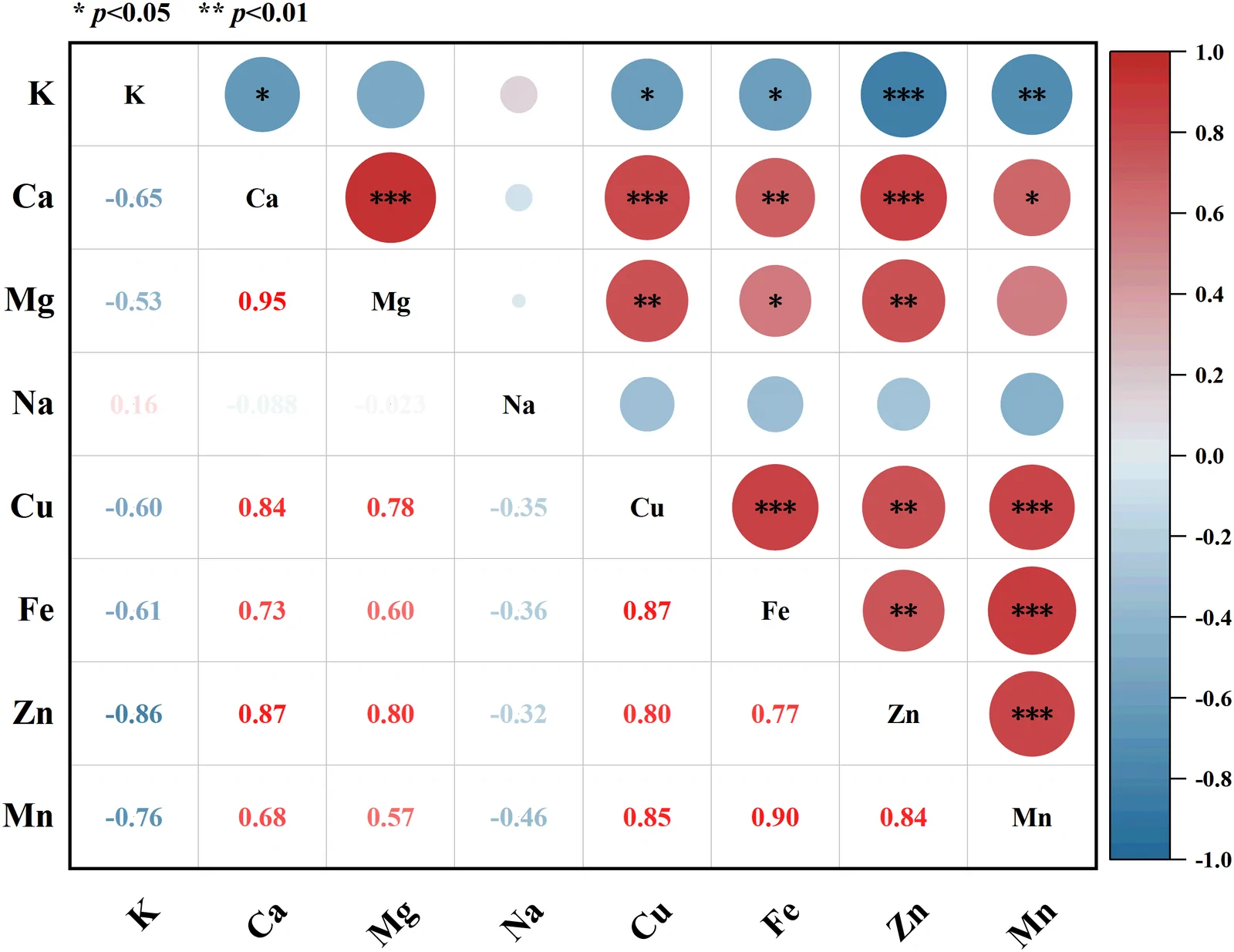
Correlation analysis of mineral elements in young shoots of *D. brandisii*. (Different tones in the image represent the positive and negative directions of correlation. Red indicates a positive correlation among mineral elements, while blue indicates a negative correlation among them. The depth of color represents the absolute value of the correlation coefficient—the darker the color, the larger the absolute value of the correlation coefficient. Correlation analysis was based on data from six biological replicates per stage and part (n = 6). *p<0.05, **p<0.01).

### 3.6 Anti-nutritional substances

Oxalic acid content differed significantly (*P < 0.01*) among growth stages and parts. As the young shoots grew, oxalic acid content in the upper part first decreased and then increased; in the middle part, it showed a continuous upward trend; in the lower part, it exhibited a pattern of decrease-increase-decrease. Oxalic acid content reached its maximum (5.90 mg·g^-1^) in the middle part at Stage IV, and its minimum (4.14 mg·g^-1^) in the upper part at Stage II (**Figure 7A**).

**Figure 7:**
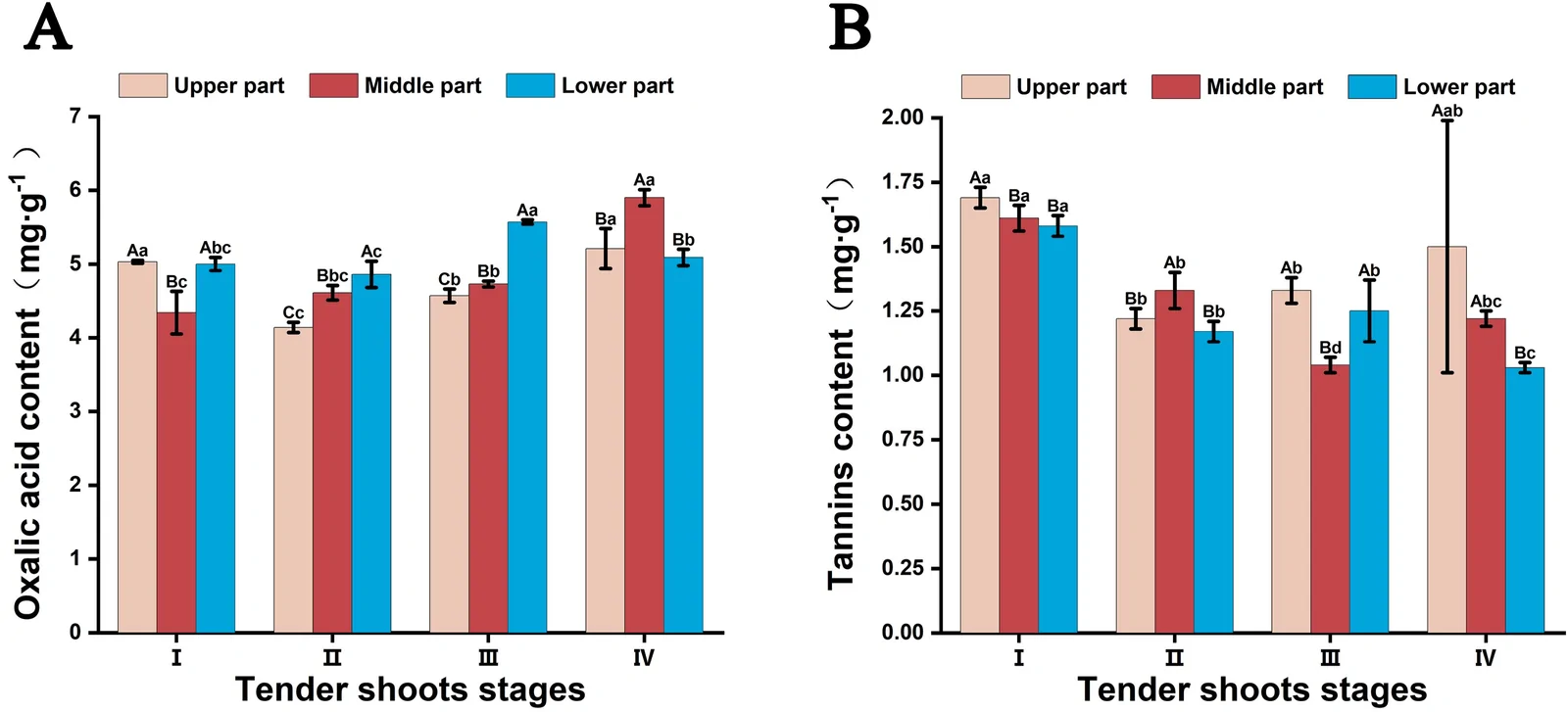
Changes of anti-nutritional substance content in different parts of young bamboo shoots at different growth stages. (The uppercase letters **(A, B)** in the upper left of the image indicate: **(A)** Oxalic acid; **(B)** Tannins. Different uppercase letters on the bars indicate significant differences among different parts within the same growth stage (*P<0.05*), while different lowercase letters on the bars indicate significant differences among different growth stages within the same part (*P<0.05*). Values are means of six biological replicates (n = 6)).

Tannin content also differed significantly (*P<0.05*) among stages and parts. In the upper and middle parts, tannin content first decreased and then increased, while in the lower part it showed a decrease-increase-decrease pattern. Tannin content peaked in the upper part at Stage I (1.69 mg·g^-1^) and then generally declined, reaching its lowest value (1.03 mg·g^-1^) in the lower part at Stage IV (**Figure 7B**). This decrease in tannin content during later growth stages is consistent with the consistently sweet taste of young *D. brandisii* shoots, which lack significant bitterness or astringency.

### 3.7 Comprehensive assessment

Indicators were classified as positive or negative according to their effects on eating quality and nutritional value, with reference to the Recommended Dietary Allowances (RDAs) and Tolerable Upper Intake Level (UL) in the Chinese Dietary Reference Intakes (DRIs, 2023) (Chinese Nutrition Society, 2023). The following were treated as positive indicators: moisture content, vitamin C, soluble sugar, protein, starch, fat, total amino acids, essential amino acids, sweet amino acids, umami amino acids, aromatic amino acids, Ca, Mg, and Na. Conversely, bitter amino acids, lignin, cellulose, flavonoids, K, Cu, Zn, Mn, Fe, oxalic acid, and tannins were treated as negative indicators. Among these, K, Cu, Zn, Mn, and Fe—although essential elements—were designated as negative indicators because their relatively high concentrations posed a potential risk of excessive intake relative to the UL values. After sum-of-squares normalization of all indicators, TOPSIS comprehensive evaluation and ranking were performed. The results showed that among all growth stages of young *D. brandisii* shoots, Stage III exhibited the best overall quality, with the highest relative closeness value (**Table 1**). Within this stage, the upper and middle parts were identified as the optimal edible portions.

**Table 1:** TOPSIS comprehensive evaluation ranking of different parts of *D. brandisii* young shoots at different stages.

Variable		Distance to positive ideal solution (D+)	Distance to negative ideal solution (D-)	Closeness coefficient (C)	Rank
Stage	Stage III	1.634	2.641	0.618	1
	Stage II	1.89	2.251	0.544	2
	Stage IV	2.551	2.475	0.492	3
	Stage I	2.51	2.415	0.490	4
Period and part	Upper part of Stage III	1.163	1.519	0.566	1
	Middle part of Stage III	1.181	1.499	0.559	2
	Middle part of Stage I	1.406	1.495	0.515	3
	Lower part of Stage III	1.423	1.507	0.514	4
	Middle part of Stage II	1.386	1.445	0.510	5
	Upper part of Stage I	1.556	1.608	0.508	6
	Upper part of Stage II	1.331	1.366	0.507	7
	Middle part of Stage IV	1.405	1.433	0.505	8
	Lower part of Stage II	1.434	1.296	0.475	9
	Lower part of Stage IV	1.734	1.553	0.473	10
	Upper part of Stage IV	1.436	1.283	0.472	11
	Lower part of Stage I	1.438	1.267	0.469	12

## 4 Discussion

### 4.1 Growth pattern of young shoots in *D. brandisii*

Plant height and basal diameter growth are co-regulated by genetic characteristics and ecological factors. This study showed that the basal diameter growth of young *D. brandisii* shoots slowed down approximately 21 days after emergence, exhibiting a “slow-fast-slow” trend, which is consistent with the general pattern of vegetative growth in most plants. The entire height growth period lasted 25 days, with a “slow-then-fast” growth rate, similar to the early growth patterns of other bamboo species such as *P. edulis*, *Bonia amplexicaulis*, and *D. sinicus* (Chen et al., 2022; Niu et al., 2022; Ma et al., 2024). Cheng et al. (2017) also reported that radial growth of young *P. edulis* shoots began to decelerate about 15 days after emergence and essentially stopped by 25 days, completing the growth cycle earlier than height growth. In addition to intrinsic genetic and developmental programs, environmental factors play important regulatory roles in bamboo shoot growth phenotypes. Although environmental factors were not directly measured in this study, existing research indicates that ambient temperature can affect the growth of *P. edulis* by regulating the expression of genes involved in cell division and elongation (Chen et al., 2022). Shoot height and diameter at breast height are significantly positively correlated with altitude (Zhang et al., 2019). Persistent drought inhibits bamboo forest growth (Ge et al., 2022), and monthly precipitation negatively affects internode diameter and wall thickness (Zhang et al., 2024). These findings suggest that the growth phenotypes of young *D. brandisii* shoots may also be modulated by similar environmental factors, a possibility that warrants further investigation.

### 4.2 Morphological-anatomical observation of internode cells in young bamboo shoots

Internode elongation in young shoots is associated with cell division and elongation in the intercalary meristem (Xiong et al., 1980). In this study, the growth rate of *D. brandisii* shoots followed a “slow-then-fast” pattern, with the average daily height increment at Stage IV being 4.78 times that at Stage I. Specifically, at Stages I and II, numerous nuclei were observed in both parenchyma and fiber cells, indicating that internode growth was dominated by cell division. In contrast, at Stages III and IV, fiber cell length increased markedly and some parenchyma cells no longer exhibited visible nuclei, signaling a transition to cell elongation as the dominant process, which drove the rapid internode growth. Dynamic changes in cell length and nuclear number within tissues provide direct evidence of cell elongation and division (Cui et al., 2012). In the present study, cells in the upper internode maintained vigorous division throughout, characterized by large nuclei and dense cytoplasm, whereas cells in the middle and lower internodes gradually elongated and their division capacity weakened from Stage II onward. These observations are consistent with those of Li et al. (2018) in *P. edulis*. Moreover, cell elongation ceased as the vascular bundles gradually differentiated and matured. Furthermore, the basic structure of internodes consists of vascular bundles embedded in parenchyma tissue, a feature consistent with that reported for several bamboo species, including *P. edulis*, *D. farinosus*, and *D. asper* (Xu et al., 2008, 2022; Chen et al., 2024b). Based on the above anatomical findings and recent innovations in sequencing technologies, the molecular mechanisms underlying rapid bamboo growth have been extensively investigated, including transcriptomics (Wei et al., 2019), genome-wide analysis of circular RNAs (Wang et al., 2019), DNA methylation analysis (Niu et al., 2022; Li et al., 2023), and subgenomic analysis of gibberellin-related genes (Mao et al., 2025). However, the relevant molecular regulatory mechanisms in *D. brandisii* remain to be further explored.

### 4.3 Study of nutrient accumulation in tender bamboo shoots

#### 4.3.1 Moisture content

In this study, the moisture content of young *D. brandisii* shoots reached its maximum (94.01%) in the upper part at Stage I and gradually decreased thereafter. This trend is consistent with observations for *C. tumidissinoda* shoots and may be attributed to continuous water consumption by physiological activities such as respiration and transpiration (Yang et al., 2015). Moreover, moisture content consistently followed the order upper > middle > lower, in agreement with findings for *C. utilis* shoots (Qin et al., 2024), which may be associated with differences in cell growth and maturation among internodes. Previous studies have demonstrated that plant water status is closely linked to cell growth, with cells achieving elongation by modulating cell wall properties and turgor pressure (Braidwood et al., 2014), and that significant differences in vacuole occupancy exist between meristematic cells and elongation zone cells (Dünser et al., 2019). Nevertheless, the precise regulatory mechanisms by which this complex system governs plant cell growth remain to be further elucidated. Notably, higher moisture content is associated with increased tenderness and palatability of bamboo shoots (Wang et al., 2009). In the present study, moisture content exceeded 90% across all stages and parts, indicating that young *D. brandisii* shoots maintain excellent eating quality throughout the entire edible period.

#### 4.3.2 Sugars content

The levels, transport, utilization, and storage of carbohydrates in plant cells are regulated by multiple factors, including environmental conditions, cellular physiological activities, and the plant developmental stage (Jeandet et al., 2022). In this study, starch content reached its maximum (1.00%) in the lower part at Stage II, while soluble sugar content peaked (9.81%) in the lower part at Stage IV. Shi et al. (2019) also observed an initial increase in starch content in young *P. prominens* shoots, consistent with our findings. This trend may be attributed to enhanced photosynthesis after shoot emergence, with the photosynthetic products temporarily stored as starch in the shoot. Subsequently, during Stages III and IV, internode cells elongated rapidly, and the shoots entered a fast-growth phase, during which starch was degraded through a series of enzymatic reactions to provide energy for rapid growth, leading to a decline in starch content (Zhu et al., 2018). In contrast, soluble sugar content showed an increasing trend. This likely results from the accumulation of sugars in internodes derived from starch degradation or transport from mature tissues during rapid shoot growth. Song et al. (2016) demonstrated that non-structural carbohydrates can be transferred from mature stems to young shoots through underground rhizomes during rapid growth, providing sufficient energy. This translocation mechanism may be one reason for the continuous increase in soluble sugar content in young shoots and warrants further investigation. Soluble sugar is the primary determinant of sweetness in bamboo shoots. In this study, soluble sugar contents were higher at Stages III and IV, indicating that the shoots exhibit superior sweetness during these two stages.

#### 4.3.3 Protein content

As the shoots grew, protein content generally increased, reaching its maximum in the lower part at Stage III, which is consistent with the observations of Huang et al. (2025b) in young *D. latiflorus* shoots. This trend can be attributed to two main factors. First, as essential structural and functional components of plant cells, substantial amounts of protein are required during vigorous shoot growth to support cell division, elongation, and material transport (Titus, 2018; Arkhipov et al., 2019). Second, emerging shoots lack functional absorptive roots, so the nitrogen required for protein synthesis is supplied mainly by the parent ramet through the interconnected rhizome system. In moso bamboo, ^15^N labeling has shown that 72.53% of shoot nitrogen at the early growth stage derives from the parent ramet (Zhang et al., 2025a), implying that a similar nitrogen-supply pathway may also support *D. brandisii* shoots. Protein is an essential nutrient for humans and plays a beneficial role in brain and nervous system development (Cohen Kadosh et al., 2021). In the present study, the protein content of young shoots was relatively high at Stages III and IV, and moderate consumption of these shoots can provide high-quality protein.

#### 4.3.4 Fat content

In this study, the fat content of young shoots gradually increased with growth, was lowest in the middle part at Stage I and reached its maximum (2.78%) in the lower part at Stage IV. Similarly, Wang et al. (2020) reported that total fat content increased significantly approximately 10 days after shoot emergence, suggesting that fat accumulates as a storage substance during the young shoot stage. Singer et al. (2016) demonstrated that light exposure promotes plant fatty acid synthesis; therefore, the increased light exposure following shoot emergence may represent an additional factor contributing to the rise in fat content. In addition, the rapid cell division and elongation during shoot growth require continuous synthesis of membrane phospholipids (Xiao et al., 2026), which may further contribute to fat accumulation, particularly in the elongating middle and lower internodes. Moreover, low fat content is a distinctive characteristic of bamboo shoots (≤3 g fat per 100 g fresh weight), rendering them suitable for consumption by individuals who are overweight, diabetic, or hyperlipidemic (Chongtham et al., 2011).

#### 4.3.5 Cellulose and lignin content

As the shoots grew, the contents of cellulose and lignin in all parts gradually increased, consistently following the order lower > middle > upper, and reached their maximum values in the lower part at Stage IV, which is consistent with the findings of Pandey and Ojha (2013) and Yang et al. (2021). As major structural components of the cell wall (Wang et al., 2012b), cellulose and lignin accumulate in close association with vigorous cell division and elongation. In the present study, cells in the upper intercalary meristem remained continuously dividing, whereas fiber cells in the middle and lower parts initially increased in number and elongated, followed by further differentiation, maturation, and lignification, resulting in significantly higher contents in the lower part than in the middle and upper parts. Moreover, studies have demonstrated that a decrease in moisture content in young shoots upregulates the expression of lignin synthesis-related genes and promotes lignin synthesis (Becerra-Moreno et al., 2015; Fan et al., 2006); therefore, differences in moisture content among the upper, middle, and lower parts may partially explain the uneven distribution of lignin, a possibility that warrants further investigation. Bamboo shoots are rich in dietary fiber, which can ameliorate obesity by modulating gut microbiota and fatty acid metabolism (Zhou et al., 2023); however, excessive lignin content increases shoot hardness, adversely affecting texture and reducing palatability (Huang et al., 2018). Consequently, owing to their lower lignin contents, the upper and middle parts remained more tender and palatable than the lower part at every stage.

#### 4.3.6 Vitamin C content

Vitamin C plays important roles in antioxidation, collagen synthesis, and the prevention of scurvy, and humans must obtain it from their diet (Gallie, 2013). Vitamin C content exhibited stage- and tissue-specific dynamics, with an overall downward trend in most parts. This decline may reflect three interacting mechanisms: (1) dilution of ascorbate by rapid cell elongation and biomass expansion; (2) continuous consumption of ascorbate as an antioxidant substrate by ascorbate peroxidase, which scavenges the reactive oxygen species generated during cell-wall lignification in the maturing lower internodes; and (3) reduced ascorbate biosynthesis as cells transition from division to elongation. Furthermore, vitamin C has poor stability and is susceptible to light, temperature, and enzymatic oxidation; changes in environmental factors after shoot emergence may also accelerate its degradation. Vitamin C loss during postharvest storage is a key factor contributing to the quality deterioration of bamboo shoots. A recent study demonstrated that low-temperature plasma-activated water treatment can effectively inhibit the respiration rate of *D. brandisii* shoots and delay the decline of vitamin C and other nutrients (Yang et al., 2026a), providing a novel strategy for maintaining postharvest quality.

#### 4.3.7 Flavonoid content

In this study, flavonoid content generally decreased with shoot growth, reaching its maximum in the upper part at Stage I, which is consistent with previous findings for *P. edulis* shoots (Guo et al., 2023). Moreover, the flavonoid content in the lower part was consistently and significantly lower than that in the upper and middle parts, similar to the trend observed in *C. utilis* shoots (Qin et al., 2024). Flavonoid biosynthesis and metabolism are co-regulated by genetic factors and environmental conditions, including temperature and light (Wang et al., 2018; Gharibi et al., 2019; Yang et al., 2020). For example, photoperiod has been shown to influence plant secondary metabolite synthesis, with long-day conditions generally increasing flavonoid accumulation to enhance photoprotection (Uleberg et al., 2012), while elevated temperatures also promote the production of secondary metabolites (Yang et al., 2018). Although flavonoids possess multiple bioactivities, such as antioxidant and anti-inflammatory properties (Chagas et al., 2022), the bitterness they impart remains a critical factor affecting food flavor. Many bamboo species develop increased bitterness after emergence and consequently require processing prior to consumption (Liu and Yu, 2024). The gradual decrease in flavonoid content during the growth of young *D. brandisii* shoots may therefore be associated with reduced bitterness, potentially contributing to their desirable sweet and tender taste. However, this hypothesis warrants further verification through quantitative analysis of bitter compounds.

#### 4.3.8 Free amino acid content

The types and contents of the individual free amino acids (FAAs) are important indicators for evaluating the nutritional and flavor value of foods, as they not only participate in flavor formation but also serve as precursors of volatile flavor compounds (Yang et al., 2020). In this study, 17 amino acids, including eight essential amino acids for humans, were detected in young *D. brandisii* shoots, indicating a relatively complete amino acid profile. The total free amino acid content exhibited a “decrease–increase–decrease” trend across the four growth stages, which may be attributed to stage-dependent regulation of carbon and nitrogen metabolism. The relatively high TAA content at Stage I likely originated from the vigorous metabolic activity of the apical meristem, where amino acids accumulated as nitrogen reserves and precursors for protein synthesis. The decrease at Stage II may be explained by the conversion of amino acids into structural proteins and the involvement of carbon skeletons in energy metabolism via the tricarboxylic acid (TCA) cycle. The transient increase at Stage III may reflect an increased demand for secondary metabolite synthesis, such as the accumulation of phenylalanine (Phe), a precursor for phenolic compounds. The subsequent decline at Stage IV may indicate a metabolic shift toward the synthesis of structural components such as cellulose and lignin. The TAA content in the upper part was consistently and significantly higher than that in the lower part, consistent with the concept that the apical region requires higher amino acid concentrations as precursors for protein synthesis (Uleberg et al., 2012). Sweet amino acids reached their highest levels in the upper part at Stage III, when the shoots were undergoing rapid growth; the accumulation of proline may promote cell elongation by regulating cellular osmotic pressure, thereby enhancing shoot palatability. In contrast, bitter amino acids peaked in the lower part at Stage III, paralleling the trend observed for lignin content, possibly due to increased activity of phenylalanine ammonia-lyase (PAL). The underlying mechanism warrants further investigation.

#### 4.3.9 Mineral element content

Mineral elements are essential for plant metabolism. In this study, the contents of macro-elements (K, Ca, Mg, Na) and trace elements (Cu, Fe, Zn, Mn) in young *D. brandisii* shoots exhibited significant spatiotemporal heterogeneity. Overall, the contents of most elements decreased with shoot growth and generally peaked at Stage I, which is closely related to the high demand for mineral nutrients during early vigorous cell division and organogenesis, followed by a “dilution effect” resulting from subsequent cell elongation and accelerated lignification. In terms of spatial distribution, a clear apical dominance effect was observed, with most elements (except Na) following the order upper > middle > lower, consistent with the nutrient transport polarity shaped by the vascular bundle development gradient. K was the most abundant macro-element, peaking in the lower part at Stage II. Potassium activates various enzymes, promotes metabolic reactions and protein synthesis (Qiao et al., 2018), and increases vacuolar solute potential to generate turgor pressure for cell elongation (Wang et al., 2012a); the K peak observed in the lower part at Stage II may be attributed to the high potassium demand during rapid elongation of the lower internodes. Ca and Mg both reached their maximum levels in the middle part at Stage I and gradually declined thereafter. As a key component of the middle lamella of the cell wall (Bou Daher and Braybrook, 2015), early accumulation of Ca facilitates cell wall construction in newly formed cells, while Mg serves as the core element of chlorophyll and a cofactor for numerous enzymes. The trace elements Fe, Zn, and Mn all peaked in the upper part at Stage I and decreased subsequently, consistent with the high demand for trace elements as enzyme cofactors in the metabolically active apical meristem.

#### 4.3.10 Anti-nutritional substance content

Oxalic acid and tannins are the main anti-nutritional substances in bamboo shoots, and their contents directly affect eating quality and safety. Oxalic acid, an important organic acid, participates in the chelation of minerals such as calcium in plants, playing a key role in maintaining cell wall structural stability and regulating mineral balance (Tong et al., 2018). In addition, oxalic acid can enhance plant responses to environmental stress. Studies have shown that oxalic acid secretion can help plants alleviate saline-alkali stress by improving photosynthetic characteristics and increasing antioxidant enzyme activity (Chen et al., 2024a). Therefore, the increase in oxalic acid in the middle part at Stage IV may be a physiological response of young shoots to environmental stress. Tannins, as a class of polyphenolic compounds, possess various biological activities such as antioxidant and antimicrobial effects, but they also affect the flavor and palatability of food. In this study, tannin content reached its maximum in the upper part at Stage I, which may indicate that early shoots require higher defensive capacity against external environmental factors such as pest and disease attacks or extreme climatic conditions. By Stage IV, tannin content in the lower part dropped to its lowest level. As shoots grow, the tannin biosynthesis pathway may be weakened or shifted toward other physiological functions, such as lignin synthesis to support structural development. Zhang et al. (2017) found that tannins are the main source of bitterness and astringency in *D. latiflorus* shoots, with their content positively correlated with bitterness intensity. This finding differs from our results but is consistent with the consistently sweet taste of young *D. brandisii*, which lack significant bitterness. The differences in the underlying regulatory mechanisms require further investigation.

### 4.4 Integrated nutrient dynamics

Coupling the anatomical framework with the nutritional data revealed a coherent developmental sequence in which metabolic shifts parallel cytological transitions. During Stages I–II, when internode growth was dominated by cell division, the high contents of moisture, minerals, amino acids, vitamins, and storage carbohydrates reflected the intense metabolic activity required for cell proliferation and primary cell-wall synthesis. During Stages III–IV, the transition from cell division to cell elongation was accompanied by a shift toward structural carbon allocation: cellulose and lignin accumulated in parallel with fiber-cell elongation and secondary wall thickening, while starch was degraded and soluble sugars accumulated to sustain respiration and cell-wall biosynthesis (Zhu et al., 2018). This coupling provides a mechanistic explanation for the TOPSIS result, as Stage III lies at the transition between the division- and elongation-dominated phases and thus combines high nutritional density with only moderate lignification, representing the optimal trade-off between nutritional value and edible texture. Furthermore, the increase in phenylalanine and other aromatic amino acids at Stage III may have provided precursors for lignin biosynthesis via the phenylpropanoid pathway (Yang et al., 2021), as reflected by the concurrent rise in lignin content in the lower part. Nevertheless, this study was conducted at a single experimental site in Nanning, Guangxi. Because shoot growth and nutrient accumulation are regulated by climatic and soil conditions, the optimal harvest window (Stage III, 19–22 d) identified here applies specifically to the climatic and soil conditions of the Nanning site and should not be directly generalized to a production standard for the species. Further multi-site validation across the main introduction regions of *D. brandisii* is therefore needed to establish region-specific harvest standards.

## 5 Conclusion

This study systematically reveals the growth patterns and dynamic changes of nutrients and chemical components in young *D. brandisii* shoots through multidimensional analyses of morphology, anatomy, and physiology. The edible growth period of young shoots lasts 25 days, with height growth following a “slow-then-fast” trend driven by both cell division and elongation. This period can be divided into four stages: Stage I (1–11 d), Stage II (12–18 d), Stage III (19–22 d), and Stage IV (23–25 d). During Stages I–II, height growth is relatively slow, dominated by cell division; during Stages III–IV, height growth is faster, dominated by cell elongation. Basal diameter growth exhibits a “slow-fast-slow” pattern. Nutrients and chemical components show significant spatiotemporal heterogeneity: moisture content, vitamin C, flavonoids, and aromatic amino acids are highest in the upper part at Stage I; starch peaks in the lower part at Stage II, whereas protein, essential amino acids, and bitter amino acids all peak in the lower part at Stage III; soluble sugar, fat, cellulose, lignin, and umami amino acids mainly accumulate during Stages III–IV; Ca, Mg, Fe, Zn, Mn, and Cu contents peak at Stage I and then decline, whereas K peaks at Stage II and Na shows no clear directional change; among anti-nutritional substances, oxalic acid reaches its highest level in the middle part at Stage IV, while tannin content peaks in the upper part at Stage I and then continuously declines. Combined with the TOPSIS comprehensive evaluation model, this study identifies Stage III as the stage with the best overall quality of young *D. brandisii* shoots, and the upper and middle parts at this stage as the optimal edible portions. This study systematically elucidates the early growth and metabolic patterns of young *D. brandisii* shoots, providing theoretical support for scientifically dividing growth stages and precisely controlling harvesting windows, as well as practical guidance for standardized cultivation, quality optimization, and sustainable bamboo vegetable production.

## Data Availability

The raw data supporting the conclusions of this article will be made available by the authors, without undue reservation.
