# Supplementary material for: Correlations among different parts, growth dynamics, anatomical structure and quality traits of Dendrocalamus brandisii young shoots
Source: Front Plant Sci. 2026 Oct 22;17:1982727. doi: 10.3389/fpls.2026.1982727 (PMC13736837; doi:10.3389/fpls.2026.1982727)
Supplement: Supplementary file 1 [file SupplementaryFile1.docx]

**Supplementary Information**

**Supplementary Figure S1** Introduction to the experimental Site and the uses of bamboo shoots as food

**Supplementary Figure S2 Material handling**

Note: A. Intact *D. brandisii* shoots; B. After removing the bracts, the young shoots were transversely sectioned into three equal segments (upper, middle, and lower parts) based on the edible portion; C. Shoots with bracts removed; D. Samples were cut into 1 cm³ cubes, fixed in FAA fixative, sealed, labeled, and stored at 4°C for subsequent laboratory analysis.

| **Supplementary Table S1** Nutrient, Chemical Components and Anti-nutritional Substances Contents of *D. brandisii* young shoots at Different Growth Stages and Parts | | | | | | | | | | | |
| --- | --- | --- | --- | --- | --- | --- | --- | --- | --- | --- | --- |
| Stage and part | Moisture content(%) | Soluble sugar(%) | Starch  (%) | Protein  (%) | Fat  (%) | Vitamin C (mg/100g) | Cellulose  (%) | Lignin  (%) | Flavonoid  (mg·g^-1^) | Oxalic acid  (mg g^-1^) | Tannins  (mg g^-1^) |
| Ⅰ- U | 94.01±0.08Aa | 0.44±0.02Bd | 0.66±0.01Cc | 3.08±0.08Ac | 1.36±0.01Bd | 5.29±1.26Aa | 2.86±0.86Aa | 2.15±0.15Bc | 5.81±0.34Aa | 5.03±0.02Aa | 1.69±0.04Aa |
| Ⅱ- U | 93.22±0.35Ab | 5.87±0.19Bc | 0.95±0.04Ba | 3.78±0.20Ab | 2.29±0.04Bc | 1.89±0.12Bd | 3.00±0.68Ba | 2.88±0.15Cb | 5.41±0.80Aa | 4.14±0.07Cc | 1.22±0.04Bb |
| Ⅲ- U | 92.85±0.60Ab | 7.54±0.45Bb | 0.89±0.04Ab | 3.96±0.17Bb | 2.33±0.05Bb | 2.95±0.37Ac | 2.85±0.70Ba | 3.05±0.13Ca | 3.84±0.35Ab | 4.57±0.09Cb | 1.33±0.05Ab |
| Ⅳ- U | 92.54±0.93Ab | 9.12±0.13Ba | 0.47±0.03Cd | 4.35±0.03Aa | 2.44±0.03Ba | 3.44±0.12Ab | 3.81±0.79Ca | 3.19±0.11Ca | 4.02±0.33Ab | 5.21±0.27Ba | 1.50±0.49Aab |
| Ⅰ- M | 93.71±0.41Aa | 0.20±0.01Cc | 0.85±0.03Aa | 2.94±0.14Ac | 1.18±0.03Cd | 3.32±0.31Ba | 3.09±0.74Ab | 2.22±0.16Bd | 5.05±0.26Ba | 4.34±0.29Bc | 1.61±0.05Ba |
| Ⅱ- M | 93.05±0.31Aa | 7.80±0.21Ab | 0.34±0.01Cd | 3.52±0.16Ab | 2.07±0.09Cc | 1.48±0.17Cd | 3.19±0.67Bb | 3.16±0.08Bc | 3.78±0.53Bb | 4.61±0.1Bbc | 1.33±0.07Ab |
| Ⅲ- M | 92.09±1.00Ab | 9.51±0.08Aa | 0.76±0.06Bb | 4.11±0.20Ba | 2.25±0.04Cb | 3.08±0.34Ab | 3.49±1.00Bb | 3.43±0.10Bb | 4.20±1.18Aab | 4.73±0.04Bb | 1.04±0.03Bd |
| Ⅳ- M | 91.64±0.84ABb | 7.98±0.25Cb | 0.56±0.02Bc | 4.15±0.16Aa | 2.38±0.01Ca | 1.85±0.36Bc | 4.80±0.98Ba | 5.20±0.09Ba | 3.75±0.94Ab | 5.9±0.11Aa | 1.22±0.03Abc |
| Ⅰ- L | 93.09±0.43Ba | 5.06±0.33Ad | 0.73±0.03Bb | 2.39±0.16Bd | 1.52±0.04Ad | 1.48±0.12Cc | 3.46±1.01Ab | 2.53±0.10Ad | 4.40±0.55Ca | 5±0.09Abc | 1.58±0.04Ba |
| Ⅱ- L | 92.12±0.19Bab | 7.96±0.31Ac | 1.00±0.04Aa | 3.82±0.12Ac | 2.46±0.04Ac | 2.09±0.37Ab | 4.67±0.96Aa | 3.99±0.11Ac | 3.47±0.24Bb | 4.86±0.18Ac | 1.17±0.04Bb |
| Ⅲ -L | 91.24±1.32Abc | 9.50±0.14Ab | 0.60±0.03Cc | 4.60±0.12Aa | 2.55±0.05Ab | 2.83±0.12Aa | 4.98±1.04Aa | 4.90±0.06Ab | 2.25±0.20Bc | 5.57±0.03Aa | 1.25±0.12Ab |
| Ⅳ-L | 90.58±0.59Bc | 9.81±0.20Aa | 0.96±0.02Aa | 4.10±0.13BAb | 2.78±0.03Aa | 1.68±0.09Bc | 5.79±0.50Aa | 7.29±0.08Aa | 3.36±0.79Ab | 5.09±0.11Bb | 1.03±0.02Bc |

Note: Ⅰ,Ⅱ,Ⅲ, andⅣ represent growth stages Ⅰ,Ⅱ,Ⅲ, and Ⅳ, respectively; U, M, and L represent upper, middle, and lower parts, respectively. Different uppercase letters indicate significant differences among different parts within the same growth stage (*P<0.05*), while different lowercase letters indicate significant differences among different growth stages within the same part (*P<0.05*). Data are presented as mean ± SD (n=6). The same as below.

| **Supplementary Table S2** F-values and P-values of nutrient, chemical and anti-nutritional contents in young shoots | | | | | | | | |
| --- | --- | --- | --- | --- | --- | --- | --- | --- |
| Content | Index | Stage Ⅰ | Stage Ⅱ | Stage Ⅲ | Stage Ⅳ | Upper part | Middle part | Lower part |
| Moisture | *F*-value | 8.945 | 20.227 | 3.124 | 7.549 | 5.932 | 8.800 | 10.237 |
|  | *P*-value | 0.003 | <0.001 | 0.073 | 0.005 | 0.005 | <0.001 | <0.001 |
| Soluble sugar | *F*-value | 1264.058 | 135.248 | 101.227 | 126.370 | 1336.358 | 3633.820 | 425.171 |
|  | *P*-value | <0.001 | <0.001 | <0.001 | <0.001 | <0.001 | <0.001 | <0.001 |
| Starch | *F*-value | 72.577 | 666.781 | 61.134 | 758.589 | 229.965 | 352.107 | 232.944 |
|  | *P*-value | <0.001 | <0.001 | <0.001 | <0.001 | <0.001 | <0.001 | <0.001 |
| Protein | *F*-value | 23.542 | 2.895 | 12.206 | 3.703 | 43.855 | 35.005 | 156.082 |
|  | *P*-value | 0.001 | 0.132 | 0.008 | 0.090 | <0.001 | <0.001 | <0.001 |
| Fat content | *F*-value | 226.824 | 56.854 | 74.339 | 421.877 | 1258.070 | 622.531 | 1236.126 |
|  | *P*-value | <0.001 | <0.001 | <0.001 | <0.001 | <0.001 | <0.001 | <0.001 |
| Vitamin C | *F*-value | 1081.500 | 21.341 | 1.184 | 222.763 | 369.923 | 191.471 | 171.571 |
|  | *P*-value | <0.001 | <0.001 | 0.333 | <0.001 | <0.001 | <0.001 | <0.001 |
| Cellulose | *F*-value | 0.709 | 8.181 | 8.377 | 9.613 | 2.184 | 5.051 | 6.836 |
|  | *P*-value | 0.508 | 0.004 | 0.004 | 0.002 | 0.122 | 0.009 | 0.002 |
| Lignin | *F*-value | 13.025 | 151.533 | 603.138 | 2847.889 | 72.782 | 754.932 | 3034.702 |
|  | *P*-value | <0.001 | <0.001 | <0.001 | <0.001 | <0.001 | <0.001 | <0.001 |
| Flavonoid | *F*-value | 18.428 | 20.025 | 12.610 | 1.225 | 23.676 | 3.386 | 18.223 |
|  | *P*-value | <0.001 | <0.001 | <0.001 | 0.321 | <0.001 | 0.038 | <0.001 |
| Oxalic acid | *F*-value | 14.836 | 25.961 | 238.953 | 17.141 | 32.200 | 52.647 | 22.241 |
|  | *P*-value | 0.005 | 0.001 | 0.001 | 0.003 | 0.001 | 0.001 | 0.001 |
| Tannins | *F*-value | 10.279 | 13.506 | 13.506 | 4.139 | 4.014 | 154.732 | 66.670 |
|  | *P*-value | 0.002 | 0.01 | 0.01 | 0.037 | 0.022 | 0.001 | 0.001 |

Note: Columns Stage Ⅰ-Ⅳ represent comparisons among different parts within the same growth stage; columns Upper part, Middle part, Lower part represent comparisons among different growth stages within the same part. *P<0.05* indicates significant difference, and *P<0.01* indicates highly significant difference. The same as below.

| **Supplementary Table S3** Dynamic changes in free amino acids and their compositional profiles in *D. brandisii* young shoots across growth stages and parts | | | | | | | | | |
| --- | --- | --- | --- | --- | --- | --- | --- | --- | --- |
| Stage and part | Asp (mg/100g) | Thr (mg/100g) | Ser (mg/100g) | Glu  (mg/100g) | Gly (mg/100g) | Ala (mg/100g) | Cys (mg/100g) | Val (mg/100g) | Met  (mg/100g) |
| Ⅰ- U | 8.86±1.53Aa | 8.18±1.42Aa | 7.22±1.20Ab | 39.73±6.85Aab | 7.01±1.29Aa | 45.12±7.61Aa | 3.01±0.64Aa | 12.42±2.15Aa | 3.29±0.69Bc |
| Ⅱ- U | 6.37±1.08Aab | 3.66±0.81Bb | 9.87±1.00ABb | 36.30±4.33Abc | 2.01±0.42Abc | 35.85±3.71Aa | 2.77±0.73Aa | 4.62±0.62Abc | 29.64±3.85Bb |
| Ⅲ- U | 9.48±2.15Aa | 7.25±1.48Aa | 19.77±2.74Aa | 50.46±7.83Aa | 3.02±0.66Ab | 40.35±6.34Aa | 2.47±0.66Aa | 7.04±1.43Ab | 25.60±5.84Cb |
| Ⅳ- U | 4.54±3.19Ab | 3.83±0.94ABb | 8.38±2.02Bb | 24.72±6.14Bc | 1.17±0.29Bc | 13.38±3.26Bb | 2.03±0.36Aa | 3.28±0.78Ac | 52.76±0.99Aa |
| Ⅰ-M | 5.49±1.15Bb | 5.90±1.29ABa | 4.51±1.51Bb | 38.80±10.16Aa | 5.53±1.05Aa | 33.77±7.24Aa | 2.53±0.48Aa | 9.39±1.89Aa | 3.11±0.83Bc |
| Ⅱ-M | 5.15±0.48Aab | 5.30±0.60Aa | 12.28±2.00Aa | 45.76±5.80Aa | 2.25±0.16Abc | 24.76±2.24Bb | 1.71±0.20Bb | 4.16±0.17Ab | 32.80±4.29Bab |
| Ⅲ-M | 8.62±1.22Aa | 4.21±0.53Ba | 12.41±1.63Ba | 42.69±5.30Aa | 1.22±0.14Bc | 23.66±2.92Bb | 2.01±0.17Aab | 3.90±0.43Bb | 41.49±3.65Ba |
| Ⅳ-M | 5.67±1.71Ab | 5.90±1.63Aa | 13.18±3.20Aa | 56.83±14.57Aa | 2.83±0.69Ab | 20.37±4.91Ab | 1.87±0.44Aab | 4.29±0.87Ab | 31.56±7.58Bb |
| Ⅰ- L | 8.19±1.75ABa | 4.32±1.18Ba | 6.64±0.94ABb | 20.94±3.70Bab | 1.97±0.47Ba | 31.89±4.66Aa | 2.52±0.60Aab | 5.74±1.08Ba | 25.21±6.39Ac |
| Ⅱ- L | 4.59±1.16Ab | 2.99±0.78Ba | 7.58±1.73Bb | 29.85±7.20Ba | 1.02±0.25Bb | 20.96±4.69Bb | 2.01±0.38ABab | 2.67±0.56Bbc | 41.18±3.73Ab |
| Ⅲ -L | 3.82±1.35Bb | 4.56±0.67Ba | 10.76±1.77Ba | 30.02±4.95Ba | 1.22±0.14Bb | 15.77±1.73Bb | 2.83±0.55Aa | 3.31±0.28Bb | 72.01±5.73Aa |
| Ⅳ-L | 2.49±0.33Ab | 3.00±0.39Ba | 7.90±0.76Bb | 13.26±0.63Bb | 0.42±0.05Bc | 6.78±0.71Bc | 1.74±0.10Ab | 1.54±0.17Bc | 31.13±4.88Bc |

| **Supplementary Table S3** Dynamic changes in free amino acids and their compositional profiles in *D. brandisii* young shoots across growth stages and parts | | | | | | | | | |
| --- | --- | --- | --- | --- | --- | --- | --- | --- | --- |
| Stage and part | Ile (mg/100g) | Leu (mg/100g) | Tyr (mg/100g) | Phe  (mg/100g) | Lys (mg/100g) | His (mg/100g) | Arg (mg/100g) | Pro (mg/100g) | TAA  (mg/100g) |
| Ⅰ- U | 8.60±1.59Aa | 17.14±3.06Aa | 12.37±2.19Aa | 14.29±2.45Aa | 24.08±4.09Aa | 7.28±1.30Aa | 5.66±1.01Ac | 7.49±1.22Aab | 231.74±40.02Aa |
| Ⅱ- U | 4.84±0.71Ab | 5.32±0.84Ab | 9.04±1.07Aab | 9.49±1.14Ab | 3.62±0.25Ab | 4.01±0.51Ab | 15.98±1.92Ab | 3.88±1.81Abc | 187.27±22.35Aab |
| Ⅲ- U | 4.06±0.90Ab | 7.33±1.81Ab | 8.11±1.94Abb | 8.70±1.05Ab | 3.61±0.60Ab | 4.53±0.73Ab | 23.18±4.01Aa | 8.93±3.22Aa | 233.89±36.82Aa |
| Ⅳ- U | 1.92±0.18Bc | 4.69±1.27Abb | 6.30±1.88Bb | 5.00±1.42Ac | 1.49±0.47Bb | 1.83±0.61Abc | 7.40±2.06Ac | 3.09±0.71Bc | 145.80±25.43ABb |
| Ⅰ-M | 5.97±1.45Aa | 13.32±3.15Aa | 10.84±2.60Aab | 12.88±3.23Aa | 19.55±4.42Aa | 7.61±1.71Aa | 3.15±1.20Bc | 5.94±1.41Aa | 188.31±43.16ABa |
| Ⅱ-M | 3.47±0.20Abc | 5.01±0.28Abb | 7.24±0.42Abab | 6.10±0.52Bb | 2.76±0.77Ab | 3.39±0.20Ab | 11.88±1.93Ba | 3.07±0.47Ab | 177.08±17.50ABa |
| Ⅲ-M | 2.49±0.15Bc | 4.87±0.69Bb | 6.65±1.03Bb | 6.10±0.84Bb | 2.02±0.25Bb | 2.72±0.42Bb | 6.03±0.83Bb | 3.97±0.71Bbc | 175.05±20.11Ba |
| Ⅳ-M | 4.56±1.09Aab | 5.61±1.38Ab | 11.53±3.62Aa | 5.77±0.51Ab | 2.97±0.60Ab | 2.99±0.80Ab | 5.12±1.25Abc | 5.06±1.46Abc | 186.10±44.27Aa |
| Ⅰ- L | 6.82±1.41Aa | 7.35±1.46Ba | 9.29±1.74Aa | 7.59±1.94Ba | 4.04±0.45Ba | 3.44±0.66Ba | 3.86±0.72Ba | 3.09±0.69Bb | 152.80±29.36Ba |
| Ⅱ- L | 3.50±0.97Ab | 3.74±0.93Ab | 6.17±1.53Ab | 4.40±1.08Bbc | 0.87±0.17Bc | 2.07±0.62Bb | 2.70±0.72Cb | 2.13±0.57Ab | 138.42±27.03Ba |
| Ⅲ -L | 1.34±0.19Cc | 6.87±0.25Ba | 9.38±0.63Aa | 6.46±0.22Bab | 1.45±0.13Bb | 2.03±0.18Bb | 2.62±0.19Bb | 5.59±0.45Ba | 180.04±18.23Ba |
| Ⅳ-L | 0.00Cc | 3.25±0.41Bb | 8.26±1.48Abab | 2.54±0.35Bc | 0.00Cd | 0.89±0.25Bc | 0.35±0.07Bc | 2.59±0.50Bb | 86.14±10.04Bb |

| **Supplementary Table S4** Variations in amino acid composition of different parts of *D. brandisii* young shoots at different growth stages | | | | | | | | | | | | | | |  |
| --- | --- | --- | --- | --- | --- | --- | --- | --- | --- | --- | --- | --- | --- | --- | --- |
| Stage and part | | Essential amino acids (EAA) (mg/100g) | | EAA/TAA  (%) | | Sweet amino acid (mg/100g) | | Umami amino acid (mg/100g) | | | Bitter amino acids (mg/100g) | | Sulfur-containing and aromatic amino acids (mg/100g) | |  |
| Ⅰ- U | | 95.27±16.62Aa | | 41.10±0.12Ab | | 75.01±12.70Aa | | 48.60±8.38Aab | | | 92.75±16.21Aa | | 29.67±5.22Aa | |  |
| Ⅱ- U | | 65.20±8.72Ab | | 34.78±0.63Bc | | 55.28±5.53Ab | | 42.67±5.41ABbc | | | 77.51±9.83Aa | | 21.30±2.95Ab | |  |
| Ⅲ- U | | 68.11±13.05Bb | | 29.03±1.04Cd | | 79.32±8.92Aa | | 59.94±9.98Aa | | | 84.04±15.53ABa | | 19.28±3.65Ab | |  |
| Ⅳ- U | | 74.80±5.73Ab | | 51.88±4.99Aa | | 29.84±7.21Bc | | 29.25±9.22Bc | | | 78.37±6.84Aa | | 13.33±3.66Ac | |  |
| Ⅰ-M | | 77.74±17.74Aa | | 41.31±0.99Aa | | 55.65±11.84ABa | | 44.29±11.20ABa | | | 74.99±17.40Aa | | 26.25±6.27Aa | |  |
| Ⅱ-M | | 62.99±4.47Aa | | 35.65±1.39Ba | | 47.65±5.25Aa | | 50.91±6.29Aa | | | 69.57±5.70Aa | | 15.05±0.91Bb | |  |
| Ⅲ-M | | 67.80±6.38Ba | | 38.79±1.00Ba | | 45.47±5.83Ba | | 51.31±6.52Aa | | | 69.61±6.69Ba | | 14.75±2.04Ab | |  |
| Ⅳ-M | | 63.65±13.33Aa | | 34.44±2.66Bb | | 47.33±11.78Aa | | 62.50±16.24Aa | | | 62.87±13.06Aa | | 19.17±4.56Aa | |  |
| Ⅰ- L | | 64.52±14.32Ab | | 42.07±1.23Ac | | 47.91±7.93Ba | | 29.02±5.44Ba | | | 64.06±13.86Ab | | 19.40±4.07Aa | |  |
| Ⅱ- L | | 61.41±8.82Ab | | 44.65±2.26Ac | | 34.68±8.02Bb | | 34.45±8.35Ba | | | 61.12±8.76Ab | | 12.57±2.97Bb | |  |
| Ⅲ -L | | 98.03±7.33Aa | | 54.55±1.64Aa | | 37.90±4.71Bab | | 33.84±6.26Ba | | | 96.09±6.84Aa | | 18.67±1.10Aa | |  |
| Ⅳ-L | | 42.35±6.35Bc | | 49.05±1.61Ab | | 20.69±2.39Bc | | 15.75±0.60Bb | | | 39.70±6.05Bc | | 12.54±1.92Ab | |  |
| **Supplementary Table S5** Variations in mineral element content of *D. brandisii* young shoots at different stages and in different parts | | | | | | | | | | | | | | | |
| Stage and part | K(mg·kg⁻¹) | | Ca(mg·kg⁻¹) | | Mg(mg·kg⁻¹) | | Na(mg·kg⁻¹) | | Cu(mg·kg⁻¹) | Fe(mg·kg⁻¹) | | Zn(mg·kg⁻¹) | | Mn(mg·kg⁻¹) | |
| Ⅰ- U | 22157.67±1626.58Cc | | 2789.76±21.58Ba | | 1276.01±39.95Ba | | 280.08±72.50Ca | | 17.18±0.37Aa | 129.21±94.91Aa | | 80.79±2.97Aa | | 162.43±3.41Aa | |
| Ⅱ- U | 55730±2008.19Cb | | 1728.24±259.52Bb | | 1214.39±30.23Ba | | 463.99±188.39Aa | | 13.72±0.69Ab | 81.99±5.07Aab | | 51.02±2.06Bb | | 124.76±0.66Ab | |
| Ⅲ- U | 67190.5±4232.74Ba | | 1881.38±246.74Ab | | 1182.39±21.30Aa | | 441.75±155.94Aa | | 13.65±1.41Ab | 101.29±38.41Aab | | 46.64±2.66Ab | | 106.58±11.53Ac | |
| Ⅳ- U | 68450±6559.86Aa | | 1198.51±349.23Ac | | 994.94±135.48Ab | | 358.81±66.52Aa | | 9.72±2.96Ac | 26.27±7.71Ab | | 35.70±7.31Ac | | 73.69±11.06Ad | |
| Ⅰ-M | 35627.67±2215.74Bd | | 3445.79±192.96Aa | | 1641.33±47.80Aa | | 397.56±60.34Ba | | 16.38±0.24Ba | 71.71±17.41ABa | | 77.22±3.76Aa | | 132.64±1.87Ba | |
| Ⅱ-M | 60872.83±2717.47Bc | | 3438.26±15.98Aa | | 1614.73±11.97Aa | | 528.71±6.83Aa | | 14.61±0.24Aa | 71.46±0.30Aa | | 55.51±0.27Ab | | 62.85±0.52Bb | |
| Ⅲ-M | 72098.67±2376.17Aa | | 1387.06±775.46ABb | | 1050.95±251.66Ab | | 428.32±237.96Aa | | 5.46±2.92Bb | 34.50±0.22Bb | | 35.73±5.23Bc | | 46.12±7.34Bc | |
| Ⅳ-M | 64812.17±2236.28Ab | | 499.40±396.46Bc | | 956.95±176.18Ab | | 449.26±192.00Aa | | 4.98±2.80Bb | 14.47±5.25Bc | | 27.98±4.84ABd | | 44.76±5.61Bc | |
| Ⅰ- L | 41488.17±2444.75Ac | | 1915.78±156.93Ca | | 1256.46±55.18Ba | | 554.30±13.99Aa | | 7.89±0.67Cb | 28.73±3.47Ba | | 56.48±6.99Ba | | 65.41±4.03Ca | |
| Ⅱ- L | 76418.67±1664.60Aa | | 1117.08±546.32Cb | | 1165.68±102.29Ba | | 348.68±207.93Aa | | 11.29±1.72Ba | 28.07±21.14Ba | | 38.07±0.51Cb | | 42.83±1.39Cb | |
| Ⅲ -L | 74842.5±3918.31Aa | | 603.74±333.99Bc | | 942.58±17.30Ab | | 413.76±155.81Aa | | 3.30±0.62Bd | 11.20±7.26Bab | | 39.13±4.41Bb | | 33.64±1.30Bc | |
| Ⅳ-L | 62617.83±2895.84Ab | | 521.08±0.83Bc | | 821.03±10.16Ac | | 552.29±54.50Aa | | 5.70±1.80ABc | 7.58±0.18Bb | | 24.87±1.15Bc | | 23.11±1.60Cd | |

| **Supplementary Table S6** F-values and P-values of individual amino acids in young shoots of *D. brandisii* | | | | | | | | |
| --- | --- | --- | --- | --- | --- | --- | --- | --- |
| Content | Index | Stage Ⅰ | Stage Ⅱ | Stage Ⅲ | Stage Ⅳ | Upper part | Middle part | Lower part |
| Asp | *F*-value | 5.085 | 5.564 | 13.798 | 1.950 | 5.066 | 3.818 | 17.352 |
|  | *P*-value | 0.051 | 0.430 | 0.006 | 0.223 | 0.030 | 0.058 | 0.001 |
| Thr | *F*-value | 9.763 | 10.604 | 13.412 | 6.047 | 15.574 | 3.633 | 4.914 |
|  | *P*-value | 0.013 | 0.011 | 0.006 | 0.036 | 0.001 | 0.064 | 0.032 |
| Ser | *F*-value | 5.594 | 9.708 | 25.417 | 5.503 | 24.919 | 8.543 | 2.835 |
|  | *P*-value | 0.043 | 0.013 | 0.001 | 0.004 | 0.001 | 0.007 | 0.106 |
| Glu | *F*-value | 8.295 | 9.111 | 15.113 | 18.083 | 9.439 | 0.676 | 9.159 |
|  | *P*-value | 0.019 | 0.015 | 0.005 | 0.003 | 0.005 | 0.591 | 0.006 |
| Gly | *F*-value | 23.232 | 18.507 | 25.434 | 23.897 | 40.893 | 33.483 | 20.711 |
|  | *P*-value | 0.001 | 0.003 | 0.001 | 0.001 | 0.001 | 0.001 | 0.001 |
| Ala | *F*-value | 23.897 | 21.164 | 36.224 | 12.505 | 23.757 | 10.173 | 38.166 |
|  | *P*-value | 0.001 | 0.002 | 0.001 | 0.007 | 0.001 | 0.004 | 0.001 |
| Cys | *F*-value | 1.843 | 5.037 | 1.509 | 3.282 | 3.371 | 8.147 | 5.358 |
|  | *P*-value | 0.238 | 0.052 | 0.279 | 0.109 | 0.075 | 0.008 | 0.026 |
| Val | *F*-value | 14.315 | 20.383 | 21.230 | 13.935 | 33.311 | 26.481 | 31.323 |
|  | *P*-value | 0.005 | 0.002 | 0.002 | 0.006 | 0.001 | 0.001 | 0.001 |
| Met | *F*-value | 33.137 | 1.791 | 37.996 | 34.235 | 84.657 | 37.984 | 34.005 |
|  | *P*-value | 0.001 | 0.246 | 0.001 | 0.001 | 0.001 | 0.001 | 0.001 |
| Ile | *F*-value | 3.991 | 6.821 | 23.554 | 38.372 | 30.684 | 12.096 | 39.739 |
|  | *P*-value | 0.079 | 0.029 | 0.001 | 0.001 | 0.001 | 0.002 | 0.001 |
| Leu | *F*-value | 13.221 | 7.871 | 5.255 | 4.848 | 34.992 | 22.010 | 20.408 |
|  | *P*-value | 0.006 | 0.021 | 0.048 | 0.506 | 0.001 | 0.001 | 0.001 |
| Tyr | *F*-value | 3.173 | 10.302 | 2.264 | 2.944 | 10.987 | 4.776 | 5.634 |
|  | *P*-value | 0.115 | 0.011 | 0.185 | 0.129 | 0.003 | 0.034 | 0.023 |
| Phe | *F*-value | 7.868 | 30.847 | 19.108 | 12.296 | 24.227 | 17.243 | 14.459 |
|  | *P*-value | 0.021 | 0.001 | 0.002 | 0.008 | 0.001 | 0.001 | 0.001 |
| Lys | *F*-value | 29.550 | 29.831 | 33.535 | 31.183 | 82.161 | 45.093 | 156.891 |
|  | *P*-value | 0.001 | 0.001 | 0.001 | 0.001 | 0.001 | 0.001 | 0.001 |
| His | *F*-value | 11.783 | 20.098 | 28.344 | 9.031 | 27.699 | 23.306 | 20.493 |
|  | *P*-value | 0.008 | 0.002 | 0.001 | 0.016 | 0.001 | 0.001 | 0.001 |
| Arg | *F*-value | 6.882 | 57.248 | 69.084 | 21.120 | 29.977 | 24.900 | 27.582 |
|  | *P*-value | 0.028 | 0.001 | 0.001 | 0.002 | 0.001 | 0.001 | 0.001 |
| Pro | *F*-value | 14.519 | 2.541 | 6.670 | 5.823 | 7.181 | 6.472 | 17.552 |
|  | *P*-value | 0.005 | 0.159 | 0.030 | 0.039 | 0.012 | 0.016 | 0.001 |
| TAA | *F*-value | 3.251 | 3.889 | 4.580 | 8.406 | 5.123 | 0.113 | 9.217 |
|  | *P*-value | 0.111 | 0.083 | 0.062 | 0.018 | 0.029 | 0.950 | 0.006 |

| **Supplementary Table S7** F-values and P-values of functional and taste categories of amino acids in young shoots of *D. brandisii* | | | | | | | | |
| --- | --- | --- | --- | --- | --- | --- | --- | --- |
| Content | Index | Stage Ⅰ | Stage Ⅱ | Stage Ⅲ | Stage Ⅳ | Upper part | Middle part | Lower part |
| Essential amino acids | *F*-value | 2.691 | 0.188 | 10.249 | 9.748 | 3.974 | 1.004 | 17.060 |
|  | *P*-value | 0.146 | 0.834 | 0.012 | 0.013 | 0.053 | 0.439 | 0.001 |
| EAA/TAA | *F*-value | 0.937 | 36.222 | 312.875 | 22.873 | 43.675 | 10.590 | 30.153 |
|  | *P*-value | 0.442 | 0.001 | 0.001 | 0.002 | 0.001 | 0.004 | 0.001 |
| Sweet amino acid | *F*-value | 4.814 | 7.975 | 32.294 | 8.389 | 18.932 | 0.720 | 9.805 |
|  | *P*-value | 0.057 | 0.020 | 0.001 | 0.018 | 0.001 | 0.568 | 0.005 |
| Umami amino acid | *F*-value | 4.224 | 4.339 | 8.775 | 14.925 | 6.893 | 1.452 | 6.509 |
|  | *P*-value | 0.072 | 0.067 | 0.017 | 0.005 | 0.013 | 0.229 | 0.015 |
| Bitter amino acids | *F*-value | 2.491 | 2.937 | 4.752 | 13.416 | 0.912 | 0.537 | 18.410 |
|  | *P*-value | 0.163 | 0.129 | 0.058 | 0.006 | 0.477 | 0.670 | 0.001 |
| Sulfur-containing and aromatic amino acids | *F*-value | 2.965 | 9.927 | 2.905 | 3.126 | 8.757 | 5.298 | 5.573 |
|  | *P*-value | 0.127 | 0.013 | 0.131 | 0.117 | 0.007 | 0.026 | 0.023 |

| **Supplementary Table S8** F-values and P-values of mineral elements in young shoots of *D. brandisii* | | | | | | | | |
| --- | --- | --- | --- | --- | --- | --- | --- | --- |
| Content | Index | Stage Ⅰ | Stage Ⅱ | Stage Ⅲ | Stage Ⅳ | Upper part | Middle part | Lower part |
| K | *F*-value | 130.679 | 147.193 | 6.951 | 2.768 | 165.402 | 262.742 | 192.171 |
|  | *P*-value | 0.001 | 0.001 | 0.007 | 0.095 | 0.001 | 0.001 | 0.001 |
| Ca | *F*-value | 113.440 | 47.453 | 6.437 | 6.793 | 27.950 | 44.475 | 15.124 |
|  | *P*-value | 0.001 | 0.001 | 0.018 | 0.016 | 0.001 | 0.001 | 0.001 |
| Mg | *F*-value | 81.420 | 63.247 | 2.701 | 2.027 | 11.001 | 21.721 | 45.982 |
|  | *P*-value | 0.001 | 0.001 | 0.121 | 0.188 | 0.001 | 0.001 | 0.001 |
| Na | *F*-value | 24.981 | 1.267 | 0.022 | 2.541 | 1.623 | 0.679 | 2.394 |
|  | *P*-value | 0.001 | 0.327 | 0.978 | 0.133 | 0.236 | 0.517 | 0.119 |
| Cu | *F*-value | 497.664 | 10.166 | 32.841 | 3.950 | 13.105 | 34.724 | 26.332 |
|  | *P*-value | 0.001 | 0.005 | 0.001 | 0.059 | 0.001 | 0.001 | 0.001 |
| Fe | *F*-value | 3.272 | 20.745 | 17.175 | 12.314 | 2.866 | 39.016 | 3.839 |
|  | *P*-value | 0.086 | 0.001 | 0.001 | 0.003 | 0.081 | 0.001 | 0.039 |
| Zn | *F*-value | 28.760 | 215.585 | 6.940 | 4.769 | 80.763 | 119.321 | 38.495 |
|  | *P*-value | 0.001 | 0.001 | 0.015 | 0.039 | 0.001 | 0.001 | 0.001 |
| Mn | *F*-value | 944.557 | 8301.44 | 96.839 | 49.425 | 81.966 | 309.512 | 231.186 |
|  | *P*-value | 0.001 | 0.001 | 0.001 | 0.001 | 0.001 | 0.001 | 0.001 |
